# Streptococcus pneumoniae, S. mitis, and S. oralis Produce a Phosphatidylglycerol-Dependent, *ltaS*-Independent Glycerophosphate-Linked Glycolipid

**DOI:** 10.1128/mSphere.01099-20

**Published:** 2021-02-24

**Authors:** Yahan Wei, Luke R. Joyce, Ashley M. Wall, Ziqiang Guan, Kelli L. Palmer

**Affiliations:** a Department of Biological Sciences, The University of Texas at Dallas, Richardson, Texas, USA; b Department of Biochemistry, Duke University Medical Center, Durham, North Carolina, USA; University of Iowa

**Keywords:** LtaS, *Streptococcus mitis*, *Streptococcus oralis*, *Streptococcus pneumoniae*, glycolipids, lipidomics, lipoteichoic acid

## Abstract

Lipoteichoic acid (LTA) is a Gram-positive bacterial cell surface polymer that participates in host-microbe interactions. It was previously reported that the major human pathogen Streptococcus pneumoniae and the closely related oral commensals S. mitis and S. oralis produce type IV LTAs. Herein, using liquid chromatography/mass spectrometry-based lipidomic analysis, we found that in addition to type IV LTA biosynthetic precursors, S. mitis, S. oralis, and S. pneumoniae also produce glycerophosphate (Gro-P)-linked dihexosyl (DH)-diacylglycerol (DAG), which is a biosynthetic precursor of type I LTA. *cdsA* and *pgsA* mutants produce DHDAG but lack (Gro-P)-DHDAG, indicating that the Gro-P moiety is derived from phosphatidylglycerol (PG), whose biosynthesis requires these genes. S. mitis, but not S. pneumoniae or S. oralis, encodes an ortholog of the PG-dependent type I LTA synthase, *ltaS*. By heterologous expression analyses, we confirmed that S. mitis *ltaS* confers poly(Gro-P) synthesis in both Escherichia coli and Staphylococcus aureus and that S. mitis *ltaS* can rescue the growth defect of an S. aureus *ltaS* mutant. However, we do not detect a poly(Gro-P) polymer in S. mitis using an anti-type I LTA antibody. Moreover, Gro-P-linked DHDAG is still synthesized by an S. mitis *ltaS* mutant, demonstrating that S. mitis LtaS does not catalyze Gro-P transfer to DHDAG. Finally, an S. mitis *ltaS* mutant has increased sensitivity to human serum, demonstrating that *ltaS* confers a beneficial but currently undefined function in S. mitis. Overall, our results demonstrate that S. mitis, S. pneumoniae, and S. oralis produce a Gro-P-linked glycolipid via a PG-dependent, *ltaS*-independent mechanism.

**IMPORTANCE** The cell wall is a critical structural component of bacterial cells that confers important physiological functions. For pathogens, it is a site of host-pathogen interactions. In this work, we analyze the glycolipids synthesized by the mitis group streptococcal species, S. pneumoniae, S. oralis, and S. mitis. We find that all produce the glycolipid, glycerophosphate (Gro-P)-linked dihexosyl (DH)-diacylglycerol (DAG), which is a precursor for the cell wall polymer type I lipoteichoic acid in other bacteria. We investigate whether the known enzyme for type I LTA synthesis, LtaS, plays a role in synthesizing this molecule in S. mitis. Our results indicate that a novel mechanism is responsible. Our results are significant because they identify a novel feature of S. pneumoniae, S. oralis, and S. mitis glycolipid biology.

## INTRODUCTION

The Gram-positive bacteria Streptococcus mitis and S. oralis, members of the mitis group streptococci, are among the major oral colonizers that protect against human gingivitis via production of hydrogen peroxide, neutralization of acids, and secretion of antimicrobial compounds (1–5). They are also opportunistic pathogens that are among the leading causes of community-acquired bacteremia and infective endocarditis (IE) (6–8). Our understanding of how these organisms colonize, survive, and interact with the human host in these different niches is incomplete and requires further mechanistic study.

Streptococcus pneumoniae also belongs to the mitis group streptococci and shares >99% identity in 16S rRNA sequence with both S. mitis and S. oralis (9, 10). S. pneumoniae mainly colonizes the mucosal surfaces of the human upper respiratory tract and is a well-known human pathogen causing pneumonia, meningitis, and otitis media, among other infections, and is a significant cause of morbidity and mortality worldwide (11, 12). Though S. mitis, S. oralis, and S. pneumoniae differ in their colonization abilities and pathogenic potential, multiple studies have shown that they share some common mechanisms of host-microbe interactions. For instance, S. mitis and S. oralis may serve as reservoirs of pneumococcal virulence-associated and antibiotic resistance genes (13–15), and immunity against S. mitis provides protection against S. pneumoniae colonization (16). We recently reported that S. mitis, S. oralis, and S. pneumoniae scavenge intermediates of human phospholipid metabolism and utilize them to synthesize the zwitterionic phospholipid phosphatidylcholine (PC), a pathway that potentially modulates human host immune responses (17, 18).

In addition to membrane phospholipids, another Gram-positive cell wall component that plays critical roles in host-microbe interactions is the lipoteichoic acid (LTA). Teichoic acid (TA) is a polymer typically consisting of either glycerophosphate (Gro-P) or ribitol-phosphate (Rbo-P) repeating units (19). Depending on its cell surface anchor, the TA polymer is either called wall teichoic acid (WTA), which is anchored to the peptidoglycan layers, or LTA, which is anchored to membrane lipids. LTAs with different chemical structures can trigger different immune responses from the host (20–22). According to their structural differences, LTAs have been grouped into five different types, among which the LTAs produced by Staphylococcus aureus (type I) and S. pneumoniae (type IV) have been extensively studied (23). Pneumococcal LTA was originally identified in 1943, and it was named F-antigen at that time due to its ability to cross-react with the Forssman antigen series (24). Its repeating unit consists of residues of 2-acetamido-4-amino-2,4,6-trideoxy-d-galactose (AATGal), d-glucose, Rbo-P, *N*-acetyl-d-galactosamine (GalNAc), and phosphocholine (25). Genes involved in the production of type IV LTA were summarized by Denapaite et al. based on genomic predictions and previous experimental studies (26). Orthologs of these genes are also present in S. oralis and S. mitis genomes, except that for most S. mitis and S. oralis strains, the glucose glycosyltransferase is substituted with a galactose glycosyltransferase (26, 27). Structural analysis of the type IV LTA produced by S. oralis strain Uo5 has confirmed the replacement of glucose residues by galactose, as well as revealed other differences relative to pneumococcal LTA in the repeating unit and branching structures (28).

S. mitis is the primary focus of the work presented here. In S. mitis, differently structured LTA-like polymers have been reported. Previously, Bergström et al. found that 39 of 77 S. mitis strains produce polysaccharide polymers detectable by monoclonal antibodies that separately target the pneumococcal type IV LTA polymer backbone and phosphocholine residues (29). Among the remaining strains, some of them lack phosphocholine, such as S. mitis SK598, which produces a pneumococcal LTA-like polymer with the choline residues being replaced by ethanolamine (29, 30). In addition, a few studies have reported detection of type I-like LTA, a Gro-P polymer, from S. mitis clinical isolates using anti-type I LTA antibodies (31–33). However, since these reports, species definitions among mitis group streptococci have been refined. A more recent reanalysis using the same detection technique did not detect type I LTA in four S. mitis strains, including the type strain S. mitis ATCC 49456 (34). However, genomic analysis supports the possibility of type I LTA synthesis in S. mitis, as S. mitis encodes an ortholog of the S. aureus type I LTA synthase gene, *ltaS* (26, 35). LtaS catalyzes the transfer of Gro-P from the membrane phospholipid phosphatidylglycerol (PG) and polymerizes the Gro-P units on a glycolipid anchor, forming type I LTA (36, 37).

The goal of our study was to determine whether S. mitis produces multiple types of LTAs and whether S. mitis *ltaS* mediates production of type I LTA, using the type strain ATCC 49456 as a model. We used normal-phase liquid chromatography (NPLC)-electrospray ionization/mass spectrometry (ESI/MS) to analyze membrane lipids in the mitis group streptococci. This technique is highly sensitive and specific and allows for the detection and characterization of LTA anchors and other LTA biosynthetic intermediates whose cellular levels are too low to be detected by conventional techniques such as thin-layer chromatography (TLC). We identified intermediates of type IV LTA synthesis in S. mitis, S. oralis, and S. pneumoniae. To our surprise, a type I-like LTA intermediate was observed not only in S. mitis, which encodes *ltaS*, but also in S. oralis and S. pneumoniae, which lack *ltaS* orthologs. Moreover, while S. mitis ATCC 49456 *ltaS* confers poly(Gro-P) synthesis when heterologously expressed in Escherichia coli and an S. aureus *ltaS*-deficient mutant, we confirm that S. mitis ATCC 49456 does not produce a polymer detectable by a type I LTA antibody. Importantly, *ltaS* contributes to S. mitis ATCC 49456 fitness, because deletion of *ltaS* impacted growth in human serum-supplemented medium. Overall, our results demonstrate that S. mitis, S. oralis, and S. pneumoniae synthesize intermediates of two structurally distinct lipid-anchored polymers, one type IV LTA, and one a Gro-P-containing polymer whose full structure remains to be determined.

## RESULTS

### Mitis group streptococci produce glycolipid intermediates of two structurally distinct LTAs.

**FIG 1:**
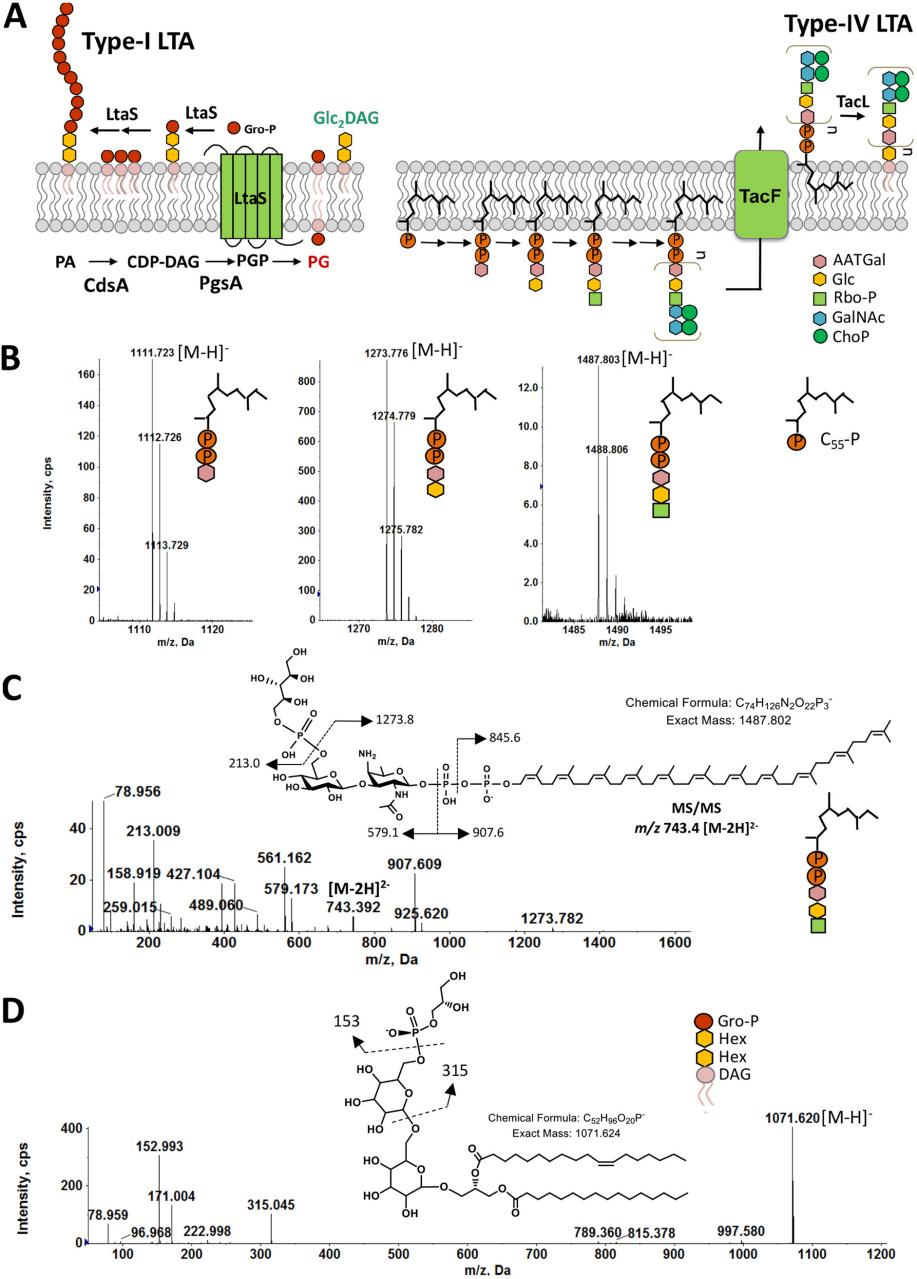
Detection of type IV LTA biosynthetic precursors and (Gro-P)-dihexosyl-DAG from the lipid extracts of S. mitis ATCC 49456 (SM61). Total lipids were extracted from S. mitis grown to mid-log phase in Todd-Hewitt broth. (A) Schematic of biosynthesis of S. aureus type I and S. pneumoniae type IV LTAs. (B) Negative ion ESI mass spectra showing the [M-H]^−^ ions of C_55_-PP-AATGal, C_55_-PP-AATGal-Gal, and C_55_-PP-AATGal-Gal-(Rbo-P). These C_55_-PP-linked saccharides are intermediates involved in assembling the pseudopentasaccharide repeating units of type IV LTA. Intensity is shown in copies per second (cps). (C) MS/MS product ion mass spectrum of the *m/z* 743.4 [M-2H]^2-^ ion of C_55_-PP-AATGal-Gal-(Rbo-P) and the MS/MS fragmentation scheme. (D) MS/MS of the *m/z* 1071.6 [M-H]^−^ ion of (Gro-P)-dihexosyl-DAG and the proposed fragmentation scheme. The chemical structures presented in panels C and D are for illustrative purposes only. The stereochemistry and linkage of hexose moieties, as well as the phosphate position on the glycerol, could not be determined by tandem MS. Abbreviations: PA, phosphatidic acid; CDP, cytidine diphosphate; PG, phosphatidylglycerol; PGP, PG-3-phosphate; Glc, glucose; C_55_-PP, undecaprenyl pyrophosphate; DAG, diacylglycerol; Gal, galacosyl; Gro-P, glycerophosphate; Rbo-P, ribitol-phosphate; AATGal, 2-acetamido-4-amino-2,4,6-trideoxy-d-galactose; GalNAc, *N*-acetyl-d-galactosamine; ChoP, phosphocholine; Hex, hexose.

LTA is usually anchored to the membrane by a saccharide-linked diacylglycerol (DAG) glycolipid (23). Structure of the glycolipid anchor varies among different LTA types, bacterial species, and even culture conditions (38). In S. pneumoniae, the pseudopentasaccharide repeating units of type IV LTA are proposed to be assembled on an undecaprenyl pyrophosphate (C_55_-PP) anchor and then transferred to a glucosyl-DAG (Glc-DAG) anchor (Fig. 1A) (25). In S. aureus, type I LTA is typically assembled on a diglucosyl-DAG (Glc_2_-DAG) anchor (Fig. 1A) (39). Listeria monocytogenes also produces type I LTA, which is linked to a galactosyl-glucosyl-DAG (Gal-Glc-DAG) anchor (40). Thus, lipid profiling has the potential to identify LTA intermediates, thereby revealing possible types of LTAs produced by a bacterium. To perform lipidomic analysis of mitis group streptococci, total lipids were extracted from bacterial cultures with a modified acidic Bligh-Dyer method and analyzed with NPLC-ESI/MS (41). We analyzed the type strain of S. mitis (ATCC 49456, referred to as SM61 hereafter), S. oralis (ATCC 35037 and the endocarditis isolate 1647), two clinically isolated S. pneumoniae strains (D39 and TIGR4), and *Streptococcus* sp. strain 1643 (referred to as SM43 hereafter), a human endocarditis isolate that was clinically identified as S. mitis but shares higher genomic identity with S. oralis (Table 1) (18, 42).

**TABLE 1:** Detection of lipoteichoic acid intermediates from selected strains of mitis group streptococci

Bacterial species	Strain	Presence of biosynthetic precursor		
		(Gro-P)-dihexosyl- DAG^*a*^	Dihexosyl- DAG	AATGal-Gal- (Rbo-P)^*b*^
S. mitis ATCC 49456 (SM61)	WT^*c*^	Yes	Yes	Yes
	*ΔcdsA*	***No***	Yes	Yes
	*ΔltaS*	Yes	Yes	Yes
*Streptococcus* sp. 1643 (SM43)	WT	Yes	Yes	Yes
	*ΔcdsA*	***No***	Yes	Yes
	*ΔpgsA*	***No***	Yes	Yes
S. oralis ATCC 35037	WT	Yes	Yes	Yes
S. oralis 1647	WT	Yes	Yes	Yes
S. pneumoniae	D39	Yes	Yes	Yes
	TIGR4	Yes	Yes	Yes

a The absence of a biosynthetic precursor is indicated by a bold italic No for emphasis.

b AATGal-Glc-(Rbo-P) in S. pneumoniae.

c WT, wild type.

Three C_55_-PP-linked intermediates of type IV LTA biosynthesis were detected in all strains analyzed. Specifically, these intermediates are C_55_-PP-linked AATGal ([M-H]^−^ at *m/z* 1111.7 of Fig. 1B, left), C_55_-PP-AATGal-Gal ([M-H]^−^ at *m/z* 1273.7 of Fig. 1B, middle, and see Fig. S1, bottom, in the supplemental material), and C_55_-PP-AATGal-Gal-(Rbo-P) ([M-H]^−^ at *m/z* 1487.7 of Fig. 1B, right). Identifications of these species are supported by the exact mass measurement and tandem mass spectrometry (MS/MS). For example, Fig. 1C shows MS/MS of the doubly deprotonated [M-2H]^2−^ ion at *m/z* 743.4 for C_55_-PP-AATGal-Gal-(Rbo-P) along with the fragmentation scheme. In addition, we also detected (Gro-P)-dihexosyl (DH)-DAG ([M-H]^−^ at *m/z* 1071.6 of Fig. 1D and Fig. S2), an intermediate that would be expected for type I LTA. The exact mass measurement (*m/z* 1071.620) is consistent with the calculated [M-H]^−^ ion mass (*m/z* 1071.624) of (Gro-P)-DHDAG containing C_16:0_ and C_18:1_ acyl chains. Furthermore, MS/MS of [M-H]^−^ ion at *m/z* 1071.6 for (Gro-P)-DHDAG (16:0/18:1) along with the fragmentation scheme are shown in Fig. 1D. The stereochemistry of the two hexoses cannot be discerned by MS/MS.

10.1128/mSphere.01099-20.3 FIG S1 Detection of type IV LTA intermediates in S. mitis ATCC 49456, S. oralis 1647, and S. pneumoniae D39. Total lipids were extracted from bacteria grown to stationary phase with a modified acidic Bligh-Dyer extraction method and analyzed with NPLC-ESI/MS in the negative ion mode. Shown are the deprotonated [M-H]^−^ ions of type IV LTA intermediates undecaprenyl pyrophosphate (C_55_-PP) (panel A, [M-H]^−^ at *m/z* 845.6) and C_55_-PP-AATGal-hexose (Hex) (panel B, [M-H]^−^ at *m/z* 1273.7). According to genomic information, the hexose moieties should be galactose in S. mitis and S. oralis and glucose in S. pneumoniae. The chemical structures presented are for illustrative purposes only. The stereochemistry of hexose moieties could not be determined by tandem MS. AATGal, 2-acetamido-4-amino-2,4,6-trideoxy-d-galactose. Download FIG S1, TIF file, 0.5 MB. Copyright © 2021 Wei et al. 2021 Wei et al. https://creativecommons.org/licenses/by/4.0/ This content is distributed under the terms of the Creative Commons Attribution 4.0 International license.

10.1128/mSphere.01099-20.4 FIG S2 Detection of glycerophosphate (Gro-P)-linked dihexosyl-diacylglycerol (DHDAG) in S. mitis ATCC 49456, S. oralis 1467, and S. pneumoniae D39. Total lipids were extracted from bacteria grown to stationary phase and analyzed with NPLC-ESI/MS in the negative ion mode. Shown are the mass spectra of the deprotonated [M-H]^−^ ions for (Gro-P)-DHDAG detected from S. mitis [panel A, retention time, ∼20.0 to 20.5 min; most abundant *m/z* 1043.6 for (Gro-P)-DHDAG (32:1)], S. oralis [panel B, retention time, ∼20.0 to 20.5 min; most abundant *m/z* 1071.6 for (Gro-P)-DHDAG (34:1)], and S. pneumoniae [panel C, retention time, ∼20.0 to 20.5 min; most abundant *m/z* 1043.6 for (Gro-P)-DHDAG (32:1)]. The acyl compositions are indicated by the numbers in parentheses; the total acyl chain carbons and double bonds are denoted by the numbers before and after the colon, respectively. Download FIG S2, TIF file, 0.7 MB. Copyright © 2021 Wei et al. 2021 Wei et al. https://creativecommons.org/licenses/by/4.0/ This content is distributed under the terms of the Creative Commons Attribution 4.0 International license.

To confirm the possible monosaccharide identity of the DAG-linked sugars, *in silico* analyses were performed to identify orthologs of known glycolipid biosynthetic genes in the genomes of the tested strains. S. pneumoniae produces the glycolipid Gal-Glc-DAG (43), for which the biosynthetic genes have been partially identified. These genes can be separated into two major groups corresponding to the biosynthetic steps they are responsible for: (i) production of nucleotide-activated sugars and (ii) transferring of the activated sugar moieties to DAG (38). As shown in Table 2, these genes include the following: confirmed UDP glucose (UDP-Glc) production gene *pgm* (encoding α-phosphoglucomutase) and *galU* (encoding UTP:α-glucose-1-phosphate uridyltransferase) (44); Leloir pathway genes that are proposed to produce UDP galactose (UDP-Gal), specifically *galK* (encoding galactokinase) and *galT2* (encoding galactose-1-phosphate uridylyltransferase 2) (45, 46); and glycosyltransferases encoded by genes Spr0982 and *cpoA* which sequentially transfer Glc and Gal residues to DAG, respectively (47, 48). S. pneumoniae R6 is an avirulent and unencapsulated derivative of S. pneumoniae D39 (49). These two strains share the same glycolipid biosynthetic genes. Using S. pneumoniae R6 as reference, orthologs of Gal-Glc-DAG biosynthetic genes with ≥87% amino acid identity were identified in the genomes of SM61, S. oralis ATCC 35037, SM43, and S. pneumoniae TIGR4 (Table 2). This analysis suggests that the DHDAG detected in our experiments is likely to be Gal-Glc-DAG.

**TABLE 2:** Orthologs of glycolipid biosynthetic genes

Chemical precursor^*a*^	Biosynthetic enzyme (reference gene^*b*^)	S. mitis ATCC 49456		S. oralis ATCC 35037		*Streptococcus* sp. 1643		S. pneumoniae TIGR4	
		Locus tag	AA^*c*^	Locus tag	AA	Locus tag	AA	Locus tag	AA
UDP-Glc	α-Phosphoglucomutase (*pgm*) (44)	SM12261_RS05265	98.6	HMPREF8579_1344	97.0	FD735_RS05500	97.2	SP_1498	100.0
	UTP: α-glucose-1-phosphate uridyltransferase (*galU*) (44)	SM12261_RS05330	95.3	HMPREF8579_0527	93.7	FD735_RS00655	93.7	SP_2092	95.7
UDP-Gal	Galactokinase (*galK*) (46)	SM12261_RS02220	97.2	HMPREF8579_1824	95.7	FD735_RS02200	97.0	SP_1853	97.5
	Galactose-1-phosphate uridyltransferase 2 (*galT2*) (46)	SM12261_RS02225	94.9	HMPREF8579_1822	93.1	FD735_RS02210	92.3	SP_1852	96.2
Glc-DAG	Glycosyltransferase (spr0982) (47)	SM12261_RS04480	96.6	HMPREF8579_1104	88.4	FD735_RS04125	88.6	SP_1076	99.3
Gal-Glc-DAG	Glycosyltransferase (*cpoA*) (48)	SM12261_RS04475	97.4	HMPREF8579_1103	87.0	FD735_RS04120	87.3	SP_1075	99.7

a Abbreviations: UDP, uridine diphosphate; Glc, glucose (glucosyl); Gal, galactose (galactosyl); DAG, diacylglycerol.

b S. pneumoniae R6 gene was used as the reference.

c Percentage of amino acid sequence identity to the referenced enzyme.

### Biosynthesis of (Gro-P)-DHDAG requires phosphatidylglycerol in mitis group streptococci.

**FIG 2:**
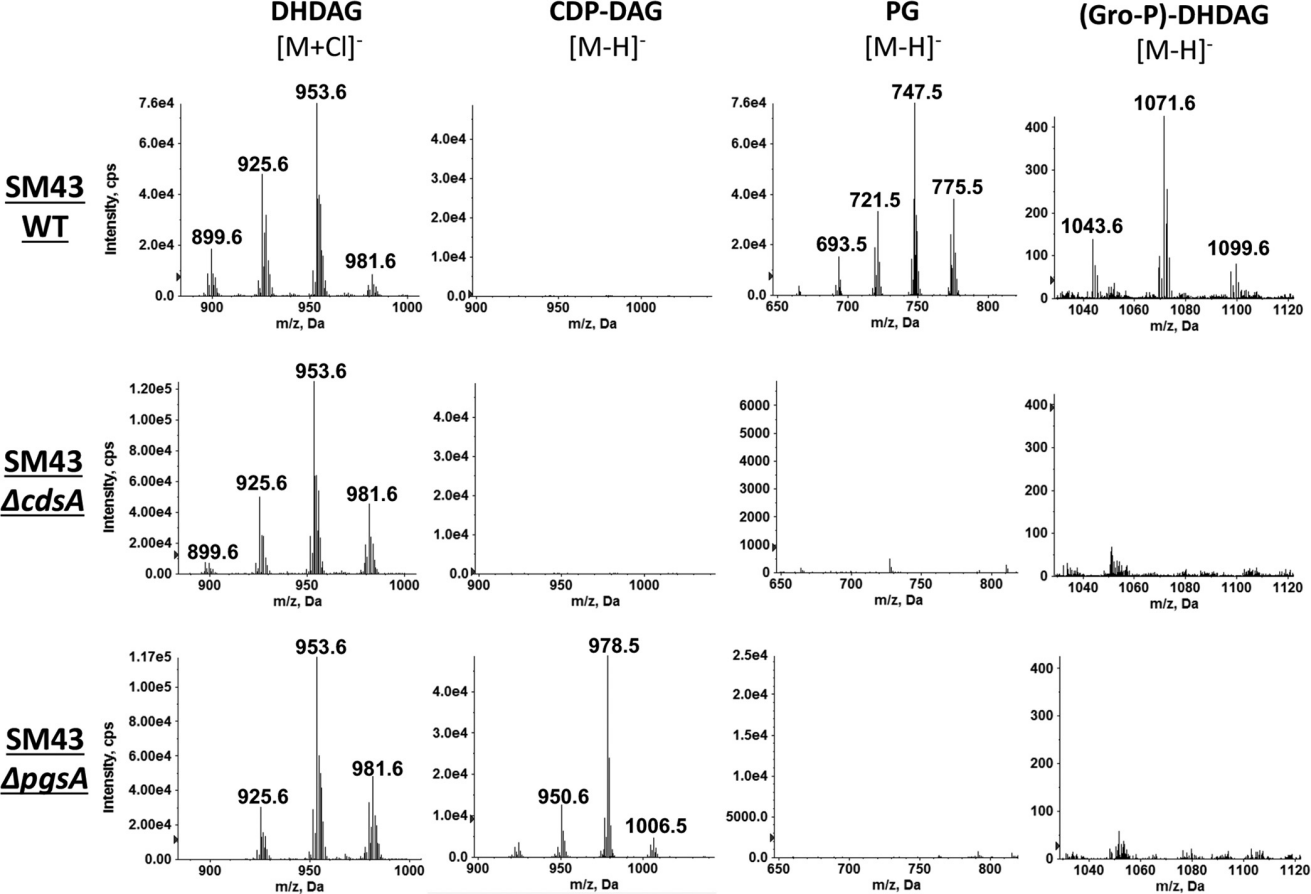
Negative ion ESI mass spectra showing the detection of phospholipids and anchor profiles from lipid extracts of wild-type (WT) *Streptococcus* sp. 1643 (SM43) and *ΔcdsA* and *ΔpgsA* strains. Total lipids were extracted from SM43 cells grown to mid-log phase in Todd-Hewitt medium. From left to right, each column correspondingly shows the mass spectra of the [M+Cl]^−^ ions of dihexosyl-diacylglycerol (DHDAG) [retention time, ∼8.0 to 10.0 min; most abundant *m/z* 953.6 for DHDAG(16:0/18:1)], [M-H]^−^ ions of CDP-DAG [retention time, ∼21.5 to 22.5 min; most abundant *m/z* 978.5 for CDP-DAG(16:0/18:1)], phosphatidylglycerol (PG) [retention time, ∼12.5 to 13.5 min; most abundant *m/z* 747.5 for PG (16:0/18:1)], and glycerophosphate (Gro-P)-linked DHDAG [retention time, ∼20.0 to 20.5 min; most abundant *m/z* 1071.6 for (Gro-P)-DHDAG(16:0/18:1)]. The identification of these lipid species is supported by both exact mass measurement and MS/MS.

In S. aureus, the Gro-P of type I LTA is produced from hydrolyzation of membrane PG (36), a process that is also required for Gro-P modification of streptococcal rhamnose-containing cell wall polysaccharides (50). To verify whether PG is the source of Gro-P for (Gro-P)-DHDAG biosynthesis in mitis group streptococci, we analyzed the lipid profiles of *cdsA* and *pgsA* mutants. The gene *cdsA* is required for the synthesis of CDP-DAG, which is then converted by PgsA to produce phosphatidylglycerophosphate (PGP), the immediate precursor of PG (Fig. 1A) (18, 41). We previously reported that *cdsA* deletion mutants of S. mitis and S. oralis do not synthesize PG, nor does a *pgsA* deletion mutant of SM43 (18, 41) (Fig. 2). Thus, lipid anchor profiles of SM43 *cdsA* and *pgsA* deletion mutants were analyzed. While the DHDAG glycolipid anchor (such as [M+Cl]^−^ at *m/z* 953.6 of Fig. 2) is observed in the wild-type, *ΔcdsA*, and *ΔpgsA* strains, the Gro-P-linked DHDAG (such as [M-H]^−^ at *m/z* 1071.6 of Fig. 2) is missing from the *ΔcdsA* and *ΔpgsA* strains. Identical anchor profiles were observed for the SM61 *cdsA* mutant (Table 1). These results demonstrate that *cdsA* and *pgsA*, or more specifically the ability to synthesize PG, are required for the biosynthesis of (Gro-P)-DHDAG in SM61 and SM43.

### S. mitis, S. oralis, and S. pneumoniae cell extracts do not react with a type I LTA antibody.

Currently, enzymes known to transfer Gro-P from PG for Gro-P polymer synthesis or Gro-P modification include the following: (i) S. aureus LtaS, the type I LTA synthase that produces poly(Gro-P) (36); (ii) L. monocytogenes LtaP, the type I LTA primase that has an overall structure and active site sequences that are very similar to those of LtaS, except that it links only the first Gro-P unit to the glycolipid anchor (35, 40); and (iii) the recently identified streptococcal Gro-P transferase GacH that links Gro-P to cell wall-attached glycopolymers (50). Bioinformatic analyses predict no orthologs of either *ltaP* or *gacH* in the genomes of the mitis group streptococci assessed here, yet an ortholog of *ltaS* is present in S. mitis as previously reported (35).

**FIG 3:**
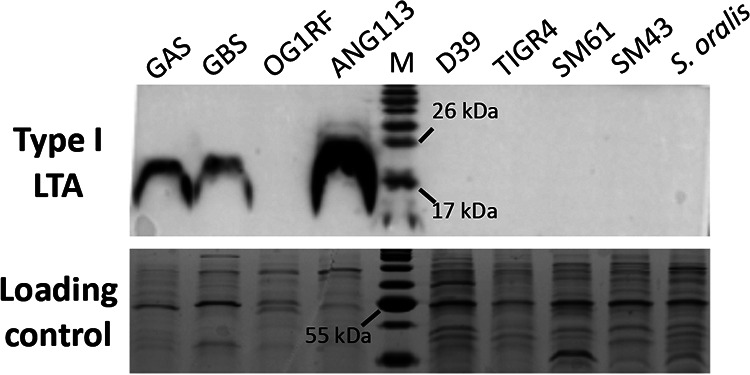
Detection of type I LTA. Cell lysates from overnight cultures of Streptococcus pyogenes NZ131 (group A streptococci [GAS]), S. agalactiae A909 (group B streptococci [GBS]), Enterococcus faecalis OG1RF, Staphylococcus aureus (ANG113), S. pneumoniae D39, S. pneumoniae TIGR4, S. mitis ATCC 49456 (SM61), *Streptococcus* sp. 1643 (SM43), and S. oralis ATCC 35037 were analyzed. Lane M contains molecular mass standards (in kilodaltons). Anti-type I LTA antibody was used to detect the production of type I LTA. Loading control was stained with Coomassie blue.

If S. mitis *ltaS* functions the same as its ortholog in type I LTA-producing bacteria like S. aureus, polymers of Gro-P will be produced and may be detectable using an anti-type I LTA antibody. Western blot analysis using a previously described anti-type I LTA antibody was conducted for SM61, SM43, S. oralis ATCC 35037, and S. pneumoniae strains. No signal was detected from cell lysates of these strains (Fig. 3) or from cell lysates of SM61 that overexpress *ltaS* in *trans* from an anhydrotetracycline-inducible vector (Fig. S3). These results are in accordance with previous observations of no immunoluminescence detection of Gro-P polymers in SM61 (34). The validity of the antibody was confirmed by positive signals detected from cell lysates of Streptococcus agalactiae, Streptococcus pyogenes, and S. aureus, all three of which produce type I LTA (Fig. 3) (36, 51, 52). Interestingly, no signal was detected from cell lysate of Enterococcus faecalis OG1RF (Fig. 3), another bacterium known to produce type I LTA (53, 54), which as reported previously is poorly recognized by the anti-type I LTA antibody (55).

10.1128/mSphere.01099-20.5 FIG S3 (A) Western blot detection of type I LTA in S. mitis ATCC 49465 (SM61) containing either the *ltaS* expression plasmid pitetR-ltaS or empty plasmid control (pitetR). Overexpression of *ltaS* was induced by the addition of 150 ng/ml anhydrotetracycline (ATC). Cell lysates were prepared from cultures grown to stationary phase. Cell lysate of S. aureus was used as positive control (+). The Western blot figure was obtained 2 min after the saturation of the positive-control signal. The loading control was stained with Coomassie blue. (B) Quantitative reverse transcription-PCR (qRT-PCR) detection of the transcript levels of *ltaS* from SM61 containing pitetR-ltaS or pitetR with or without ATC induction. Total RNA was harvested from mid-log-phase cells exposed to ATC for 30 min. Relative expression levels of *ltaS* were normalized to that of 16S rRNA of the same sample with the ΔΔ*C_T_* method. Four biologically independent replicates were obtained for each sample. Statistical analysis was performed with one-way ANOVA; an asterisk indicates 0.01 < *P* value < 0.05, and “n.s.” indicates that the values were not significant (*P* value > 0.05). Download FIG S3, TIF file, 0.3 MB. Copyright © 2021 Wei et al. 2021 Wei et al. https://creativecommons.org/licenses/by/4.0/ This content is distributed under the terms of the Creative Commons Attribution 4.0 International license.

### S. mitis LtaS mediates production of poly(Gro-P) in an E. coli heterologous host.

**FIG 4:**
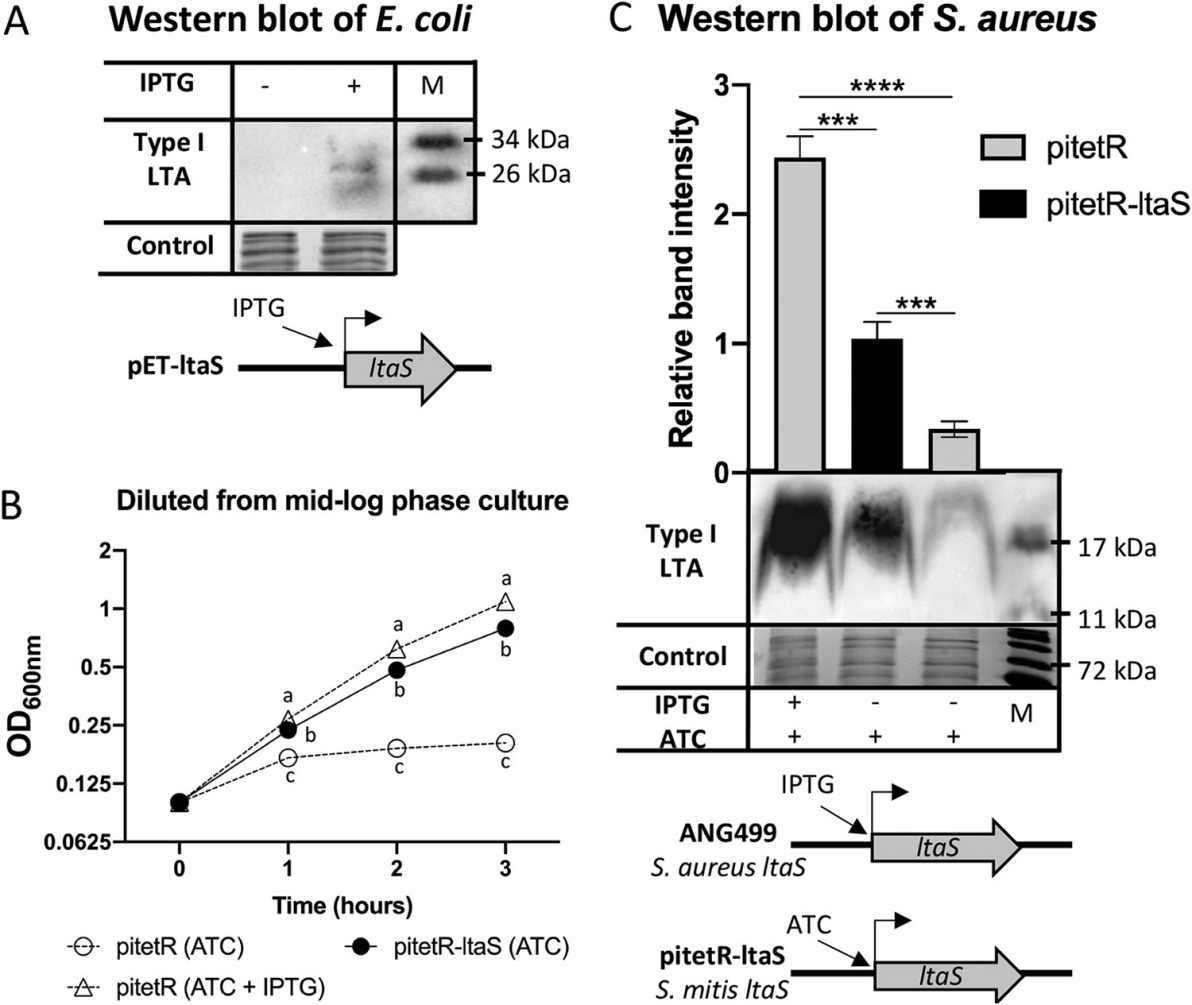
Heterologous expression of S. mitis *ltaS* in E. coli and S. aureus. (A) Western blot detection of Gro-P polymers from E. coli containing plasmid pET-ltaS grown in liquid Luria-Bertani (LB) medium with the addition (+) of isopropyl-β-d-1-thiogalactopyranoside (IPTG) and without (-) IPTG. IPTG was added to mid-log-phase bacterial cultures followed by another 30-min incubation at 37°C before cell pelleting. Three biological independent replicates were performed for each sample. (B) Growth curves of S. aureus ANG499 containing either pitetR or pitetR-ltaS grown in tryptic soy broth (TSB) with the addition of either 150 ng/ml anhydrotetracycline (ATC) only or 150 ng/ml ATC and 0.5 mM IPTG as indicated. Samples were grown in TSB with 0.5 mM IPTG overnight, followed by subculturing into fresh TSB with the indicated addition of induction reagents and incubated for 3 h. Then, another subculturing to an OD_600_ of 0.1 with fresh media same as the previous incubation was performed. After the second subculture, OD_600_ values were measured every hour and plotted. (C) Western blot detection of type I LTA from S. aureus ANG499 containing either pitetR or pitetR-ltaS. Samples were grown in the same way as described above for panel B, after the first subculturing and incubation, cells equal to 1 ml of OD_600_ at 1.2 were harvested, followed by lysate preparation and immunodetection. Schematics of induction expression of chromosomal or plasmid-carried *ltaS* were shown in both panels A and C. Loading controls of both panels A and C were stained with Coomassie blue. Western blot band intensity in panel C was normalized to the loading control and the pitetR-ltaS sample. For panels B and C, four biological replicates were performed; averages of the sample values were plotted with the error bars depicting standard deviations. Statistical analyses were performed with one-way analysis of variance (ANOVA); significant difference was determined by *P* value of <0.05. For panel B, at a given time point, the a, b, and c letters each represent a statistical group that is significantly different from other groups; *P* values of all group comparisons are <10^−6^. Statistical significance for panel C: ***, 10^−5^ > *P* value > 10^−6^; ****, *P* value < 10^−6^.

For the following analyses, the S. mitis type strain ATCC 49456 (SM61) was used as a model, and its *ltaS* ortholog (SM12261_RS03435) was renamed *ltaS*. We heterologously expressed S. mitis *ltaS* in E. coli to verify the function of the gene. This approach was previously used in studies of S. aureus *ltaS* (36). Plasmid pET-ltaS (Table 3) was constructed so that the expression of S. mitis *ltaS* could be induced with isopropyl-β-d-1-thiogalactopyranoside (IPTG) in E. coli. As shown in Fig. 4A, with the addition of IPTG, detectable bands produced by anti-type I LTA antibody targeting were observed for E. coli (pET-ltaS), demonstrating that S. mitis *ltaS* is sufficient to mediate the production of poly(Gro-P).

**TABLE 3:** Bacterial strains and plasmids used in this research

Species and strain or plasmid	Feature(s)	Reference or source
Escherichia coli		
DH5α	Cloning strain	73
K-12 MG1655	Model E. coli strain	74
BL21(DE3) pLys	Engineered E. coli strain for protein expression, contains Tn*10* that produces T7 polymerase and plasmid pLys; the presence of pLys is maintained with 5 μg/ml chloramphenicol	Novagen
Streptococcus mitis		
ATCC 49456 (SM61)	Type strain of S. mitis	ATCC
SM61 *ΔcdsA*	SM61 with coding region of *cdsA* (SM12261_RS08390) deleted	This study
SM61 *ΔltaS*	SM61 with coding region of *ltaS* (SM12261_RS03435) replaced with gene *ermB*	This study
*Streptococcus* sp.		
1643 (SM43)	Mitis group *Streptococcus* isolated from infective endocarditis patient	42
SM43 *ΔcdsA*	SM43 with coding region of *cdsA* (FD735_RS08600) deleted	18
SM43 *ΔpgsA*	SM43 with coding region of *pgsA* (FD735_RS09695) replaced with gene *ermB*	18
Streptococcus oralis		
ATCC 35037	Type strain of S. oralis	ATCC
1647	Isolated from infective endocarditis patient	42
Streptococcus pneumoniae		
D39	Clinically isolated strain, serotype 2	75
TIGR4	Clinically isolated strain, serotype 4	76
Streptococcus pyogenes		
NZ131	Clinically isolated strain, serotype M49	ATCC
Streptococcus agalactiae		
A909	Isolated from a septic human neonate, serotype Ia	ATCC
Staphylococcus aureus		
ANG113	Strain RN4220, isogenic wild-type control of ANG499	36
ANG499	Generated from strain RN4220 (wild type), expression of chromosomal *ltaS* is induced with 1 mM IPTG; the genotype maintained with 5 μg/ml erythromycin	36
Enterococcus faecalis		
OG1RF	Rifampin- and fusidic acid-resistant derivative of a human oral cavity isolate	77

Plasmids		
pABG5	Low-copy-number shuttle plasmid; confers kanamycin resistance	68
pitetR-ltaS	pABG5 with S. mitis *ltaS* coding region under control of tetracycline-inducible promoter P*_xyl/tet_*	This study
pitetR-SAltaS	pABG5 with S. aureus *ltaS* (SAV0719) coding region under control of promoter P*_xyl/tet_*	This study
pitetR	pitetR-ltaS that lacks the *ltaS* coding region and has EcoRI introduced; serves as empty plasmid control	This study
pET-28a(+)	Expression plasmid; confers kanamycin resistance	Novagen
pET-ltaS	pET-28a(+) with S. mitis *ltaS* under control of IPTG-inducible promoter	This study
pMSP3535	Confers erythromycin resistance; used to obtain *ermB* gene	7

### S. mitis *ltaS* complements an S. aureus *ltaS* mutant for type I LTA production.

In S. aureus, LtaS is required for proper cell division and efficient cell growth at 37°C (36, 56). To further confirm the physiological function of S. mitis *ltaS* in Gram-positive cells, we expressed it in a previously reported S. aureus strain that has its native *ltaS* gene under the control of an IPTG-inducible promoter (strain ANG499). Without IPTG, ANG499 is deficient for type I LTA production and has a growth defect when cultured at 37°C (36, 56). S. mitis *ltaS* was introduced into strain ANG499 by the plasmid pitetR-ltaS (Table 3), which has the S. mitis *ltaS* coding region under the control of the tetracycline-inducible promoter P*_xyl/tet_*. Addition of anhydrotetracycline (ATC) induces expression of S. mitis *ltaS*. Note that we included ATC in all experimental cultures described below, because we observed an ATC-dependent growth defect that confounded direct comparison of cultures grown in the presence or absence of ATC (Fig. S4).

10.1128/mSphere.01099-20.6 FIG S4 S. mitis *ltaS* complements the function of S. aureus *ltaS*. Single colonies of S. aureus ANG499 strain containing either pitetR-ltaS or pitetR were grown overnight in tryptic soy broth (TSB) followed by dilution into 0.1 of OD_600_ with fresh TSB with the indicated addition of 150 ng/ml ATC, 0.5 mM IPTG, no addition (No), or both 150 ng/ml ATC and 0.5 mM IPTG (ATC + IPTG). Values of OD_600_ were measured every hour for the first 3-h incubation (A and C). Then, cells equal to 1 ml of a sample with an OD_600_ of 1.2 were harvested from each sample for Western blot analysis (E and F); at the same time, another dilution identical to that described above was performed, followed with continued incubation for another 3 h and measurement of OD_600_ values every hour (B and D). For panel E, band intensities were normalized to that of sample pitetR-ltaS (ATC) and loading control. Data shown as plots were obtained from four biologically independent replicates. Statistical analyses were performed with one-way ANOVA. Significant differences (*P* value < 0.05) were shown as follows: **, 10^−3^ < *P* value < 10^−2^; ****, *P* value < 10^−6^. Values that were not significantly different are indicated by “n.s.” For panel D, at time point 1, 2, and 3, statistically different groups were indicated separately by the a, b, and c letters. *P* values between each group are all <10^−6^. Download FIG S4, TIF file, 0.6 MB. Copyright © 2021 Wei et al. 2021 Wei et al. https://creativecommons.org/licenses/by/4.0/ This content is distributed under the terms of the Creative Commons Attribution 4.0 International license.

**FIG 5:**
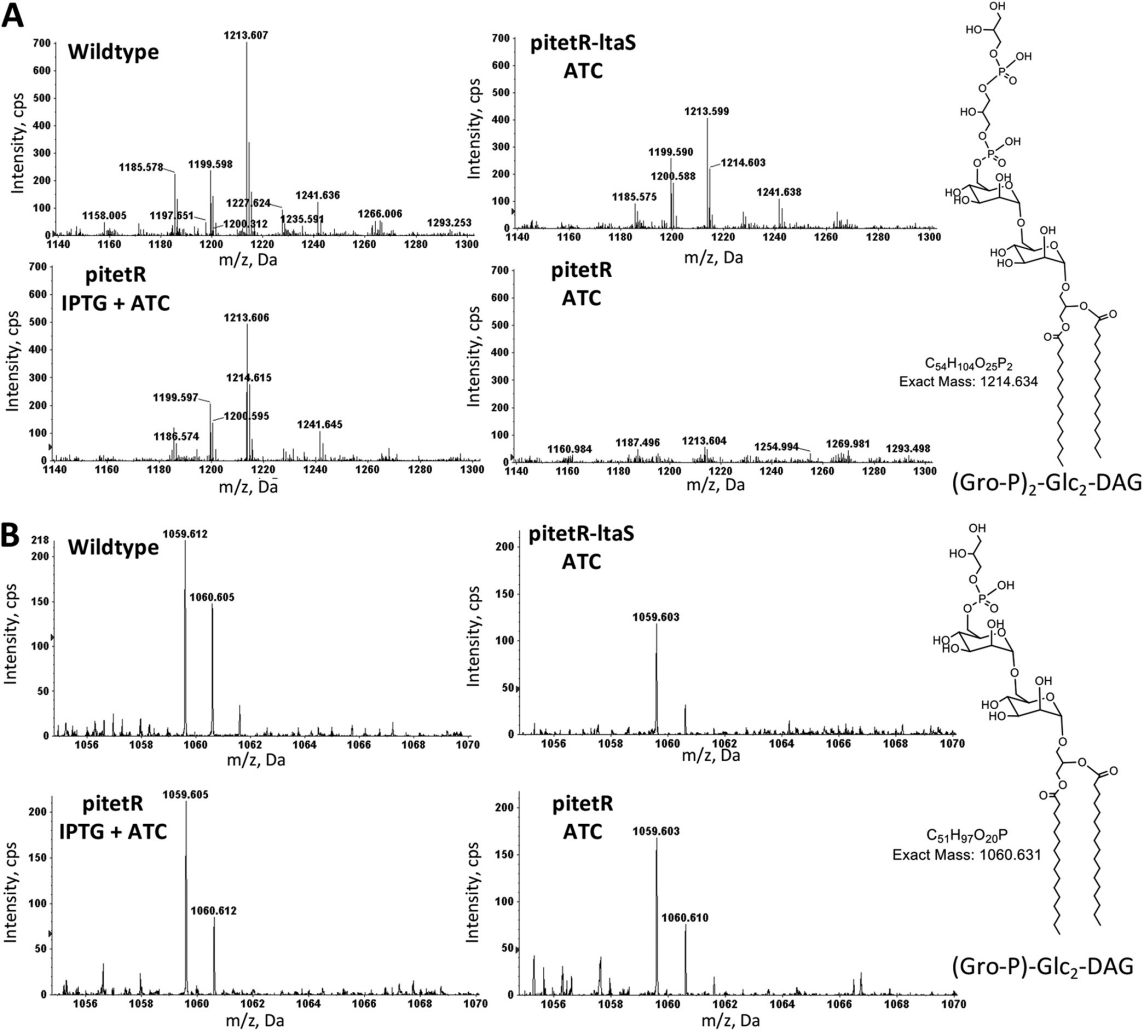
MS detection of type I LTA biosynthetic precursors that contain one or two Gro-P units in the lipid extracts of S. aureus. S. aureus strain ANG113 (wild type), ANG499 containing plasmid pitetR-ltaS (pitetR-ltaS), and ANG499 containing the vector control pitetR (pitetR) were grown in liquid tryptic soy medium to late exponential phase with the addition of ATC and IPTG as indicated. Total lipids were extracted and analyzed with NPLC-ESI/MS in the negative ion mode. Shown are the mass spectra of the deprotonated [M-H]^−^ ions for (Gro-P)-Glc_2_-DAG (retention time, ∼20.0 to 20.5 min; most abundant *m/z* 1059.6) (A) and (Gro-P)_2_-Glc_2_-DAG (retention time, ∼22.5 to 23.0 min; most abundant *m/z* 1213.6) (B). The chemical structures presented are for illustrative purposes only. The stereochemistry and linkage of hexose moieties, as well as the phosphate position on the glycerol, could not be determined by tandem MS. Three biologically independent replicates were performed for each strain under each indicated culture condition. Abbreviations: Gro-P, glycerophosphate; Glc, glucosyl; DAG, diacylglycerol.

As expected, strain ANG499 with the empty plasmid vector pitetR grew more slowly and reached a lower final optical density at 600 nm (OD_600_) value when cultured without IPTG compared to with IPTG (Fig. 4B). As expected, type I LTA production by S. aureus LtaS was induced by IPTG, confirmed by Western blot analysis (Fig. 4C) and detection of type I LTA intermediates (Gro-P)_2_-Glc_2_-DAG ([M-H]^−^ ion at *m/z* 1214.6 of Fig. 5A) and alanine-linked (Gro-P)_2_-Glc_2_-DAG ([M-H]^−^ ion at *m/z* 1285.7 of Fig. S5). Strikingly, the growth of ANG499 was also rescued by the expression of S. mitis *ltaS* from pitetR-ltaS (Fig. 4B), and type I LTA production was observed, as shown by Western blot (Fig. 4C) and lipidomic analysis (Fig. 5A and Fig. S5). These data demonstrate that S. mitis *ltaS* can complement the function of S. aureus *ltaS* and promote production of type I LTA in S. aureus. Surprisingly, (Gro-P)-Glc_2_-DAG ([M-H]^−^ ion at *m/z* 1059.6 of Fig. 5B) was detected at comparable levels from all S. aureus cultures, including the natively *ltaS*-deficient strain in the absence of IPTG induction.

10.1128/mSphere.01099-20.7 FIG S5 MS detection of type I LTA intermediate that contains two Gro-P units with one Ala modification in the lipid extracts of S. aureus. S. aureus wild-type (A) and native *ltaS* deficient strain containing either plasmid pitetR-ltaS (pitetR-ltaS) (B), or empty vector control (pitetR) (C and D) were incubated to stationary phase in tryptic soy broth with the addition of either 150 ng/ml anhydrotetracycline (ATC) or 150 ng/ml ATC with 0.5 mM IPTG as indicated. Total lipids were extracted by a modified acidic Bligh-Dyer extraction method and analyzed with NPLC-ESI/MS in the negative ion mode. Shown are the deprotonated [M-H]^−^ ions of type I LTA intermediate containing two Gro-P units with one Ala modification (retention time, ∼23.5 to 24.0 min; most abundant *m/z* 1284.6). The chemical structure presented is for illustrative purposes only. The stereochemistry and linkage of hexose moieties, as well as the phosphate position on the glycerol, could not be determined by tandem MS. Abbreviations: Ala, alanine; Gro-P, glycerophosphate; Glc, glucosyl; DAG, diacylglycerol. Download FIG S5, TIF file, 0.1 MB. Copyright © 2021 Wei et al. 2021 Wei et al. https://creativecommons.org/licenses/by/4.0/ This content is distributed under the terms of the Creative Commons Attribution 4.0 International license.

### Expression of S. aureus *ltaS* does not confer detectable type I LTA signals in S. mitis.

**FIG 6:**
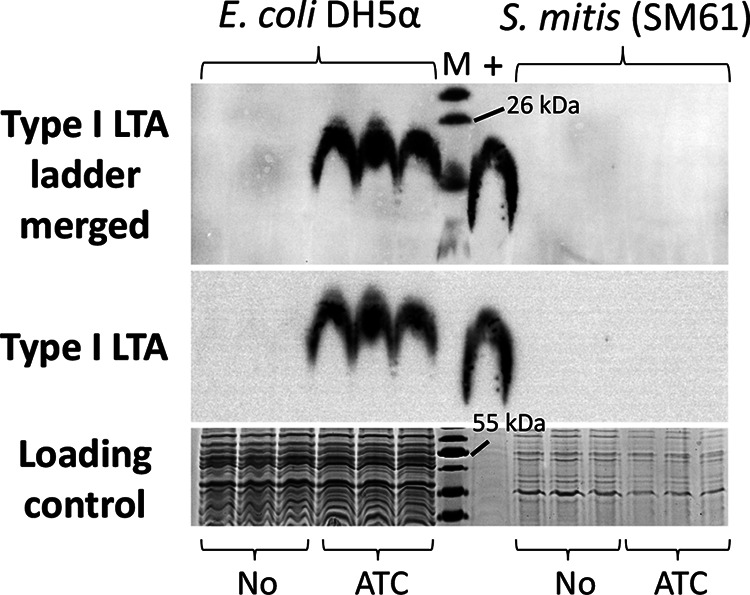
Heterologous expression of S. aureus *ltaS* in E. coli and S. mitis. E. coli DH5α and S. mitis ATCC 49456 (SM61) with pitetR-SAltaS were subcultured into media with no inducing agent (No) or with 150 ng/ml anhydrotetracycline (ATC). Cell lysate of S. aureus was used as a positive control (+). Western blot signal was obtained for 6-min cumulative exposure. The loading control was stained with Coomassie blue. Three biologically independent replicates are shown for each condition.

To test whether S. aureus *ltaS* can mediate poly(Gro-P) production in S. mitis, S. aureus *ltaS* was introduced into S. mitis with the plasmid pitetR-SAltaS. Similar to pitetR-ltaS, pitetR-SAltaS encodes S. aureus *ltaS* under the control of the ATC-inducible promoter P*_xyl/tet_*. Type I LTA production was detected by Western blot analysis for E. coli (pitetR-SAltaS) induced with ATC (Fig. 6). However, no signals were observed for S. mitis (pitetR-SAltaS) induced with ATC. In addition, lipidomic analysis detected no further structure beyond a single Gro-P linked to DHDAG for S. mitis (pitetR-SAltaS) induced with ATC.

### S. mitis lacking *ltaS* has increased serum susceptibility.

To investigate functions of *ltaS* in S. mitis, *ltaS* was deleted and exchanged for the erythromycin resistance marker *ermB*, generating S. mitis *ΔltaS*. Of note, (Gro-P)-DHDAG was still detected in the S. mitis *ΔltaS* strain, demonstrating that LtaS is not required for the addition of the Gro-P unit to the DHDAG (Table 1).

Unlike S. aureus, which requires *ltaS* for efficient growth, deletion of *ltaS* in S. mitis does not confer a growth defect under laboratory culturing conditions. Specifically, when growing in Todd-Hewitt broth at 37°C, the doubling time of the *ΔltaS* strain is 39.8 (±3.7) min, which is not significantly different from the 40.2 (±3.5) min doubling time of wild-type S. mitis (Fig. S6). Considering that the growth deficiency of S. aureus lacking *ltaS* could be mitigated by culturing at a lower temperature (56), the growth of wild-type S. mitis and *ΔltaS* strains cultured at a higher temperature was measured to determine whether the *ltaS* mutant was compromised for temperature-related stresses. The temperature 42°C was chosen as a representative of fever. Both wild-type and *ΔltaS* strains exhibited slower growth at 42°C compared to 37°C; however, no significant difference in growth rate was observed between the strains (46.2 [±3.0] and 47.4 [±3.8] min doubling times for the wild-type and *ΔltaS* strains, respectively). Moreover, no difference in susceptibilities to antibiotics targeting peptidoglycan biosynthesis, membrane integrity, and protein synthesis were observed (see Table S1 in the supplemental material). Thus, under these laboratory culture conditions, *ltaS* is not essential for the growth of S. mitis.

10.1128/mSphere.01099-20.1 TABLE S1 E-test results of the wild-type and *ΔltaS* strain of S. mitis ATCC 49456 (SM61). Download Table S1, PDF file, 0.1 MB. Copyright © 2021 Wei et al. 2021 Wei et al. https://creativecommons.org/licenses/by/4.0/ This content is distributed under the terms of the Creative Commons Attribution 4.0 International license.

10.1128/mSphere.01099-20.8 FIG S6 Growth curve of S. mitis under different culture conditions. Single colony of either S. mitis ATCC 49456 (SM61) wild type (WT) or *ΔltaS* strain cultured overnight in either Todd-Hewitt broth (THB) or chemically defined medium (CDM) were diluted into fresh indicated medium to an OD_600_ value of 0.1, followed by incubation at either 37°C (blue lines) or 42°C (red lines) as indicated in panels A and C. Values of OD_600_ were measured at incubation time of 0, 2, 4, 6, 8, and 24 h. Doubling time shown in panels B and D was calculated using the OD_600_ values acquired at incubation time of 2, 4, and 6 h. Data presented are mean values from either at least four (THB) or two (CDM) biological replicates, with standard deviations represented by the error bars. Statistical analyses were performed with one-way ANOVA; significant difference was determined by *P* value of < 0.05. Values that were not significantly different statistically are indicated by “n.s.” FIG S6, TIF file, 0.3 MB Copyright © 2021 Wei et al. 2021 Wei et al. https://creativecommons.org/licenses/by/4.0/ This content is distributed under the terms of the Creative Commons Attribution 4.0 International license.

**FIG 7:**
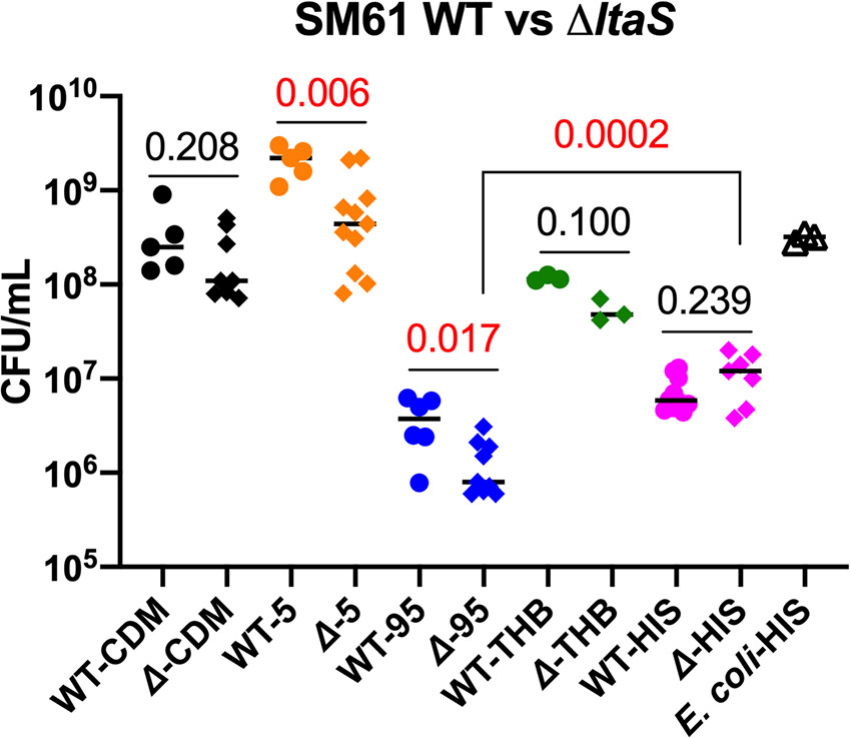
Deletion of *ltaS* alters the responses of S. mitis ATCC 49456 (SM61) to human serum. Wild-type (WT) or *ΔltaS* SM61 (Δ) strains were cultured in chemically defined medium (CDM), CDM with 5% human serum (5), 95% human serum (95) with 5% phosphate-buffered saline (PBS), Todd-Hewitt broth (THB), and 95% heat-inactivated human serum (HIS) with 5% PBS. The CFU/ml of cultures after 8-h incubation are shown. The CFU/ml of E. coli K-12 MG1655 grown in 95% human serum is below the detection limit (10^5^; not shown in figure); the CFU/ml of E. coli cultured in HIS is shown. Each symbol represents the value for one biological independent repeat. Statistical analysis was performed with the Mann-Whitney method. *P* values are indicated above the line. Statistical significance was defined by *P* value of <0.05, and significant *P* values are shown in red.

In addition, a potential role for *ltaS* in host-microbe interactions was investigated. As an oral commensal, the environment S. mitis colonizes is exposed to human gingival crevicular fluid, which is an extrudant of serum with lower concentrations of complement (57). Moreover, when invading the bloodstream and causing bacteremia and infectious endocarditis, S. mitis is constantly exposed to blood. Thus, human serum is a useful medium component for laboratory reconstruction of the host growth conditions. Supplementation of human serum into chemically defined medium (CDM) promotes the growth of S. mitis compared to nonsupplemented CDM (Fig. 7). Deletion of *ltaS* does not confer a significant difference in growth in Todd-Hewitt broth or unsupplemented CDM but does result in a significant growth deficiency in human serum-supplemented CDM, and makes S. mitis more sensitive to the killing effect of complete serum (Fig. 7). In addition, bacterial colony forming units (CFU)/ml counts were significantly higher for the *ΔltaS* mutant cultured in heat-inactivated serum compared to the mutant cultured in complete serum (Fig. 7); this significant difference was not observed for the wild-type strain. These results suggest that although *ltaS* is not required for growth of S. mitis under laboratory conditions, it confers protection against heat-sensitive serum components. Further investigation is needed to elucidate such interactions.

## DISCUSSION

In this work, we used NPLC-ESI/MS to analyze the glycolipid profiles of S. mitis, S. oralis, and S. pneumoniae strains. For all of the tested strains, biosynthetic intermediates of two structurally different LTAs were detected (Fig. 1 and Table 1). First, consistent with literature, the biosynthetic intermediates of the type IV LTA were detected, which is in agreement with genomic analysis of the biosynthetic genes (26). The second distinct LTA is indicated by the detection of (Gro-P)-DHDAG, which is similar to type I LTA polymers and unexpected based on previous reports, and thus has been the focus of this study.

On the basis of the results of genomic analysis, we proposed that the newly identified (Gro-P)-DHDAG is structured as (Gro-P)-Gal-Glc-DAG. The glycolipid Gal-Glc-DAG has been reported as the dominant glycolipid species in S. pneumoniae, and our prediction is in accordance with this previous report (43). However, the full pathway for Gal-Glc-DAG synthesis has not been fully experimentally verified in the mitis group streptococci; the stereochemistry of the hexoses requires further confirmation with structural analysis, such as with nuclear magnetic resonance (NMR).

The PG-dependent (Gro-P)-DHDAG biosynthetic process in S. mitis was then investigated, which led to the main focus of this study, functional verification of S. mitis *ltaS*. Through heterologous expression, we confirmed that S. mitis *ltaS* could directly synthesize Gro-P polymers in both E. coli and S. aureus. However, it appeared that S. mitis LtaS functions somewhat differently from S. aureus LtaS, as the expression of S. mitis *ltaS* does not fully complement the growth deficiency and the amount of type I LTA produced (Fig. 4B and C), which is not unexpected considering that S. mitis and S. aureus LtaS share only 38% sequence identity (26).

We did not detect a Gro-P polymer in wild-type S. mitis using Western blot analysis. Explanations as to why we could not detect the polymer include the following. (i) S. mitis does not produce the Gro-P polymer; instead, (Gro-P)-DHDAG is the complete and final product. (ii) A very small amount of the Gro-P polymer is produced under the culture conditions investigated here. (iii) Unique structural modifications on the Gro-P polymer hinder antibody recognition. (iv) LtaS acts on a different substrate than (Gro-P)-DHDAG in S. mitis. Further large-scale purification and structural analysis of the (Gro-P)-DHDAG-containing polymer produced by mitis group streptococci are required.

Interestingly, heterologous expression of S. aureus *ltaS* in S. mitis does not confer poly(Gro-P) production detectable by either Western blot or lipidomic analysis. It is possible that, as suggested above, unique structural modifications on the Gro-P polymer hinder antibody recognition or that S. aureus LtaS lacks the appropriate substrate(s) in S. mitis to catalyze type I LTA synthesis, in which case further studies about the substrate recognition and binding activities of S. aureus and S. mitis LtaS are needed. Last but not the least, it is possible that the canonical LtaS enzymatic function of producing poly(Gro-P) is inhibited in S. mitis.

The findings that (Gro-P)-DHDAG is still present in S. mitis *ΔltaS*, as well as in S. oralis and S. pneumoniae, which are species that do not carry genes that encode any orthologs of *ltaS*, suggest the existence of an unknown PG-dependent Gro-P transferase in these species that is responsible for the synthesis of (Gro-P)-DHDAG. Unexpectedly, (Gro-P)-Glc_2_-DAG is also seen in S. aureus deficient for *ltaS*, suggesting that an unidentified Gro-P biosynthetic enzyme(s) or biological process(es) may exist in S. aureus as well, but this is more speculative.

In other Gram-positive pathogens that synthesize type I LTA, LtaS and its product, LTA, are essential for proper cell division (40, 56, 58, 59). Inhibiting the function of LtaS is effective in extending the survival of S. aureus-infected mice (60) and sensitizing multidrug-resistant E. faecium to antibiotics (61). Though S. mitis *ltaS* is not essential for proper growth of the bacterium in normal laboratory media or for synthesizing (Gro-P)-DHDAG, it does provide some advantage to S. mitis when human serum is present in the culture media and protects against the heat-sensitive serum components.

In summary, we provide evidence that a type I-like LTA might coexist with type IV LTA in S. mitis, S. oralis, and S. pneumoniae and queried the role of *ltaS* in this process in a model S. mitis strain. To our knowledge, there is only one previous report which documents a bacterial species producing two structurally different LTAs, in Streptococcus suis, an invasive pathogen of pigs (62). Our lipidomic and genomic studies show that we have an incomplete understanding of glycolipids, LTAs, and LtaS function in mitis group streptococci and their potential roles in host-microbe interactions.

## MATERIALS AND METHODS

### Bacterial strains and growth conditions.

Unless indicated otherwise, E. coli was grown in Luria-Bertani (LB) medium, *Streptococcus* strains were grown in Todd-Hewitt (TH) medium (BD Biosciences) with S. pneumoniae grown in TH medium supplemented with 0.5% yeast extract (BD Biosciences), and E. faecalis and S. aureus were grown in tryptic soy (TS) medium (BD Biosciences). All bacterial cultures were incubated at 37°C, unless otherwise noted. Streptococci were cultured with 5% CO_2_. Chemically defined medium (CDM) was made as previously described, with the addition of 0.5 mM choline (63). Human serum-supplemented medium was made by adding complete human serum (Sigma-Aldrich) to CDM to a final concentration of 5% (vol/vol). Antibiotic concentrations were as follows: kanamycin, 50 μg/ml in E. coli, 250 μg/ml in S. aureus, and 500 μg/ml in S. mitis; erythromycin, 50 μg/ml in E. coli. Transcription of genes controlled by the promoter P*_xyl/tet_* was induced with anhydrotetracycline (ATC) at a final concentration of 150 ng/ml. Isopropyl-β-d-1-thiogalactopyranoside (IPTG)-inducible expression was mediated by the addition of IPTG to a final concentration of 1 mM. Bacterial strains and plasmids used in this research are listed in Table 3.

### Sequence analysis.

Orthologs of glycolipid biosynthetic genes were identified through using the BLASTp function against the NCBI database (64). Specifically, genes of S. pneumoniae R6 (NCBI accession no. NC_003098.1) were used as reference. The encoded amino acid sequences were input into BLASTp to search against the nonredundant protein database of S. mitis ATCC 49456 (taxid: 246201), S. oralis ATCC 35037 (taxid: 655813), *Streptococcus* sp. 1643 (taxid: 2576376), and S. pneumoniae TIGR4 (taxid: 170187) individually. The *ltaS* (SM12261_RS03435) ortholog in S. mitis ATCC 49456 was identified similarly, with the amino acid sequence of S. aureus LtaS (SAV0719) (36) being the reference. Orthologs were determined by query coverage of >95% and E value of <10^−120^.

### Mutant generation.

Deletion of *cdsA* (SM12261_RS08390) in S. mitis ATCC 49456 was conducted as previously described (65, 66). Briefly, approximately 2-kb flanking regions on either side of *cdsA* were amplified using Phusion polymerase (Thermo Fisher). PCR products were digested with restriction enzyme XmaI (New England Biolabs) and ligated with T4 DNA ligase (New England Biolabs). Ligated products were amplified using primers 61cdsA_Up_F and 61cdsA_Dwn_R (see Table S2 in the supplemental material), followed by gel extraction with the QIAquick gel extraction kit (Qiagen) per the manufacturer’s instruction. The linear construct was transformed into S. mitis via natural transformation as described previously (66). The *ΔcdsA* mutant was selected with 35 μg/ml daptomycin and confirmed by Sanger sequencing (Massachusetts General Hospital DNA Core) of the PCR product of the *cdsA* deletion region.

10.1128/mSphere.01099-20.2 TABLE S2 Primers used in this research. Download Table S2, PDF file, 0.04 MB. Copyright © 2021 Wei et al. 2021 Wei et al. https://creativecommons.org/licenses/by/4.0/ This content is distributed under the terms of the Creative Commons Attribution 4.0 International license.

Deletion of *ltaS* in S. mitis ATCC 49456 was conducted similarly with some slight modifications. Specifically, a 1-kb DNA fragment containing *ermB* was generated through PCR amplification using plasmid pMSP3535 as the template (67). Then, splicing by overlap extension PCR was performed to produce a 5-kb amplicon that sequentially contained a 2-kb fragment upstream of *ltaS*, a 1-kb *ermB*-containing fragment in reverse orientation, and a 2-kb fragment downstream of *ltaS*. The PCR product was analyzed on a 0.8% agarose gel and extracted using the QIAquick gel extraction kit (Qiagen) per the manufacturer’s instruction. Transformation of the 5-kb amplicon into S. mitis was performed as described previously (66). The *ΔltaS* mutant was selected with 20 μg/ml erythromycin and confirmed with Illumina genome sequencing (UTD Genome Core Facility).

### Plasmid construction.

Plasmids used in this research are listed in Table 3 with description of their functions. All primers used in this research are listed in Table S2.

The shuttle plasmid pABG5 was used for heterologous gene expression in Gram-positive bacteria (68). Specifically, the DNA fragment containing the S. mitis *ltaS* coding region was amplified using primers LtaS_F and LtaS_R, and the pABG5 plasmid backbone was linearized through PCR using primers pABG5-5 and pABG5-3. Gibson assembly was conducted per the manufacturer’s instructions (NEBuilder HiFi DNA assembly master mix; New England Biolabs), followed by transformation of the product into E. coli DH5α. The pABG5 with *ltaS* insert was further linearized with primers YW55 and YW56 and ligated with an 848-bp DNA fragment via Gibson assembly, producing the plasmid pitetR-ltaS. The 848-bp fragment contained a tetracycline-controlled promoter P*_xyl/tet_* and the tetracycline repressor gene *tetR* in reverse orientation. Insertion of this 848-bp fragment immediately upstream of the *ltaS* coding region makes *ltaS* expression inducible by ATC addition. The sequence of the 848-bp fragment was obtained from plasmid pRMC2 in the Addgene sequence database (69, 70), and the fragment was synthesized commercially (Integrated DNA Technologies). Induced production of the target gene *ltaS* was confirmed by Western blotting. The empty vector control pitetR was constructed via linearization of pitetR-ltaS with PCR using primers YW58 and YW59, followed by Gibson assembly for gap closure. The removal of the *ltaS* coding region was confirmed by Sanger sequencing (Massachusetts General Hospital DNA Core). Plasmid pitetR has an EcoRI site inserted after the P*_xyl/tet_*-controlled ribosomal binding site. ATC induction of S. aureus *ltaS* is mediated by plasmid pitetR-SAltaS. Specifically, an amplicon containing the S. aureus *ltaS* (SAV0719) coding region was obtained via PCR using primers YW72 and YW73, followed by Gibson assembly of this amplicon with linearized pitetR generated via EcoRI digestion. Successful insertion was confirmed with Sanger sequencing (Massachusetts General Hospital DNA Core), and the confirmed construct was transformed into E. coli DH5α for expression analysis. Plasmid pET-ltaS that mediates isopropyl-β-d-1-thiogalactopyranoside (IPTG)-inducible overexpression of *ltaS* was generated through insertion of the *ltaS* coding region immediately after the IPTG-inducible promoter region of pET-28a(+) (Novagen). Successful insertion was confirmed with Sanger sequencing (Massachusetts General Hospital DNA Core). The confirmed construct was transformed into E. coli BL21(DE3) pLys for expression analysis.

### Antibiotic susceptibility testing.

Antibiotic susceptibility testing was performed according to the bioMérieux Etest protocol with slight modifications. Specifically, a single colony of either the wild-type S. mitis ATCC 49456 or *ΔltaS* strain was selected from cation-adjusted Mueller-Hinton (MH) (BD Bacto) agar cultures, inoculated into 1 ml of MH broth, and incubated for 6 to 8 h at 37°C with 5% CO_2_. Then, 2 ml of fresh MH broth was added to the 1-ml culture, and incubation was resumed. After overnight incubation, the OD_600_ of the cultures were measured, and samples having an OD_600_ value of <0.2 were excluded from the following experimental procedures. Cultures were spread onto prewarmed MH agar plates with sterile cotton-tipped applicators, and plates were air dried for 15 to 20 min inside a biosafety cabinet. Then, Etest strips (Etest by bioMérieux) prewarmed to room temperature were applied to the plates with aseptic technique. The plates were incubated overnight at 37°C with 5% CO_2_. The MIC was determined by the intersection of the zone of inhibition with the Etest strip. At least three biological independent replicates were performed for each antibiotic-strain combination.

### Western blot analysis.

Detection of type I LTA via Western blot analysis was performed as previously described (39, 71).

For E. coli, single colonies were grown overnight in LB broth with appropriate antibiotics, followed by dilution to an OD_600_ of 0.1 with fresh media into two replicates. For E. coli DH5α containing pitetR-SAltaS, ATC was added to one set of cultures to a final concentration of 150 ng/ml, followed by 3-h incubation at 37°C before cell harvest. For E. coli BL21(DE3) pLys containing pET-ltaS, diluted bacterial cultures were incubated at 37°C for 3 h, and then IPTG was added to one set of cultures to a 1 mM final concentration, followed by another 30-min incubation at 37°C before cell harvest. To harvest cells, culture densities were normalized to an OD_600_ of 0.6, and 1 ml was pelleted, washed, resuspended in 100 μl of 2× Laemmli sample buffer, and boiled for 15 min. Boiled samples were stored at –20°C prior to electrophoretic analysis.

For S. aureus, single colonies of each S. aureus strain were grown overnight in TS broth with 0.5 mM IPTG, 5 μg/ml erythromycin, and 250 μg/ml kanamycin, and then subcultured to an OD_600_ of 0.1 into fresh TS broth containing 5 μg/ml erythromycin, 250 μg/ml kanamycin, and either 150 ng/ml ATC or 150 ng/ml ATC with 0.5 mM IPTG. After 3-h incubation, the OD_600_ was measured, and cells equivalent to 1 ml of 1.2 OD_600_ were pelleted. Cell pellets were washed and resuspended with 1 ml phosphate-buffered saline (PBS), followed by five cycles of bead-beating at 6.5 m/s for 45 s, with 5 min on ice between cycles (FastPrep-24; MP Biomedicals). After centrifugation at 200 × *g* for 1 min, cell lysates were collected, followed by pelleting at 17,000 × *g* for 10 min. The material was resuspended in 100 μl of 2× Laemmli sample buffer (Bio-Rad) followed by boiling for 15 min in a heating block.

For streptococci and E. faecalis, unless indicated, OD_600_ values of the overnight cultures were measured, followed by pelleting of cells equivalent to 1 ml of 1.2 OD_600_. Induction of *ltaS* overexpression in S. mitis was conducted similarly as in S. aureus. Specifically, overnight cultures of S. mitis containing pitetR-ltaS, pitetR, or pitetR-SAltaS were diluted to an OD_600_ value of 0.1 into fresh TH broth with 150 ng/ml ATC. After 7-h incubation, cells equivalent to 1 ml of a culture with an OD_600_ of 1.2 were harvested. All cell pellets were washed and resuspended with 1 ml PBS, then followed with the same cell disruption and lysate preparation processes as described above for S. aureus samples.

Separation of cell lysate materials are conducted through sodium dodecyl sulfate-polyacrylamide gel electrophoresis (SDS-PAGE). Specifically, 15 μl of each boiled sample was loaded onto a 15% SDS-PAG gel, followed by electrophoresis at consistent 100 voltage and subsequent polyvinylidene difluoride (PVDF) membrane transfer at consistent 350 mA. The blocking solution was PBS containing 0.05% (wt/vol) Tween 20 and 10% (wt/vol) nonfat milk; antibody solutions were PBS with 0.05% (wt/vol) Tween 20 and 5% (wt/vol) nonfat milk. For S. aureus samples, 3 μg/ml human IgG (Sigma) was added to the blocking and antibody solutions to block the activity of protein A. Primary antibody targeting type I LTA (clone 55; Hycult Technology) and secondary antibody (horseradish peroxidase [HRP]-conjugated anti-mouse IgG; Cell Signaling) were used at dilutions of 1:2,500 and 1:5,000, respectively. After adding HRP substrate (Immobilon Western; Millipore) and shaking at room temperature for 3 min, chemiluminescence signals were detected with the ChemiDoc touch imaging system (Bio-Rad) with default chemiluminescence settings. Relative band intensity was analyzed with the Image Lab Software (Bio-Rad).

### Lipidomic analysis.

Extraction of total lipids from stationary-phase cells was performed by acidic Bligh-Dyer extraction as previously described (18). Specifically, cells were grown to stationary phase in at least 5 ml of medium, followed by collection and storage at –80°C until lipid extraction with the acidic Bligh-Dyer methods. The dried lipid extracts were dissolved in 100 μl of chloroform-methanol (2:1, vol/vol). Typically, 10 μl of the dissolved solution were injected for LC/MS analysis. NPLC-ESI/MS of lipids was performed as previously described (41, 72) using an Agilent 1200 quaternary LC system (Santa Clara, CA) coupled to a high-resolution TripleTOF5600 mass spectrometer (Sciex, Framingham, MA). An Ascentis Si high-performance liquid chromatography (HPLC) column (5 μm; 25 cm × 2.1 mm; Sigma-Aldrich) was used. Mobile phase A consisted of chloroform-methanol-aqueous ammonium hydroxide (800:195:5, vol/vol/vol). Mobile phase B consisted of chloroform-methanol-water-aqueous ammonium hydroxide (600:340:50:5, vol/vol/vol/vol). Mobile phase C consisted of chloroform-methanol-water-aqueous ammonium hydroxide (450:450:95:5, vol/vol/vol/vol). The elution program was as follows: 100% mobile phase A was held isocratically for 2 min and then linearly increased to 100% mobile phase B for 14 min and held at 100% mobile phase B for 11 min. The LC gradient was then changed to 100% mobile phase C for 3 min and held at 100% mobile phase C for 3 min, and finally returned to 100% mobile phase A over 0.5 min and held at 100% mobile phase A for 5 min. Instrumental settings for negative ion ESI and MS/MS analysis of lipid species were as follows: ion spray voltage (IS) = −4,500 V; current gas (CUR) = 20 lb/in^2^ (pressure); gas-1 (GS1) = 20 lb/in^2^; declustering potential (DP) = −55 V; and focusing potential (FP) = −150 V. The MS/MS analysis used nitrogen as the collision gas. Data acquisition and analysis were performed using the Analyst TF1.5 software (Sciex, Framingham, MA).

### Serum survival test.

Overnight cultures of S. mitis were pelleted and washed with PBS, followed by subculturing into different media to an OD_600_ of 0.1. Cultures were incubated at 37°C with 5% CO_2_ for 8 h. At *t* = 0 and *t* = 8 h of incubation, bacterial CFU were quantified by serial dilution and plating on TH agar. E. coli K-12 MG1655 was prepared in a similar way described above and subcultured into 95% complete human serum (Sigma-Aldrich) and 95% heat-inactivated human serum to confirm the presence and absence of bactericidal activity, respectively. E. coli CFU were quantified by serial dilution and plating on LB agar.
