# Supplementary figures and images for: Streptococcus pneumoniae, S. mitis, and S. oralis Produce a Phosphatidylglycerol-Dependent, ltaS-Independent Glycerophosphate-Linked Glycolipid
Source: mSphere. 2021 Feb 24;6(1):e01099-20. doi: 10.1128/mSphere.01099-20 (PMC8544892; doi:10.1128/mSphere.01099-20)

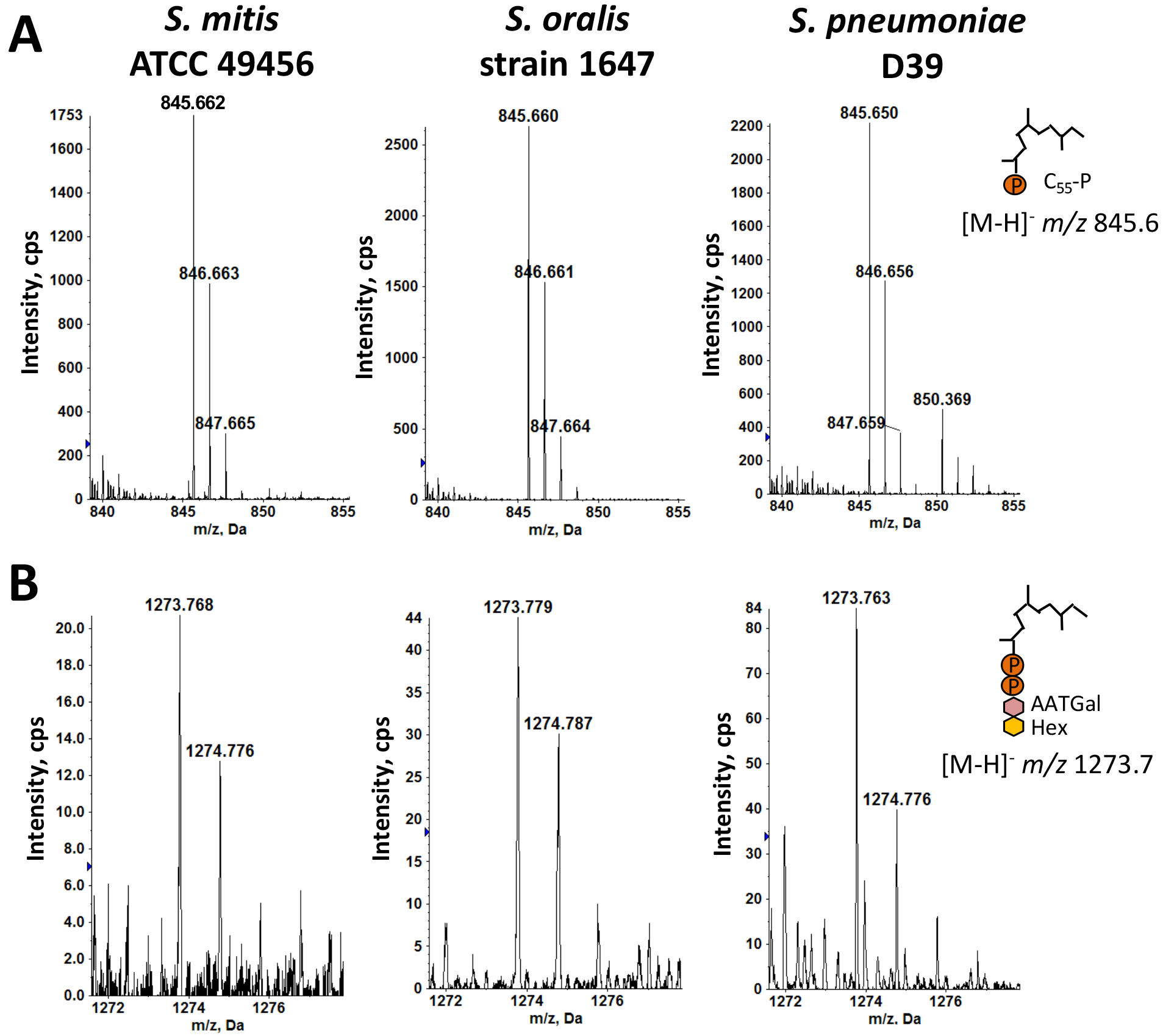

Supplement: FIG S1 [file msphere.01099-20-sf001.tif]

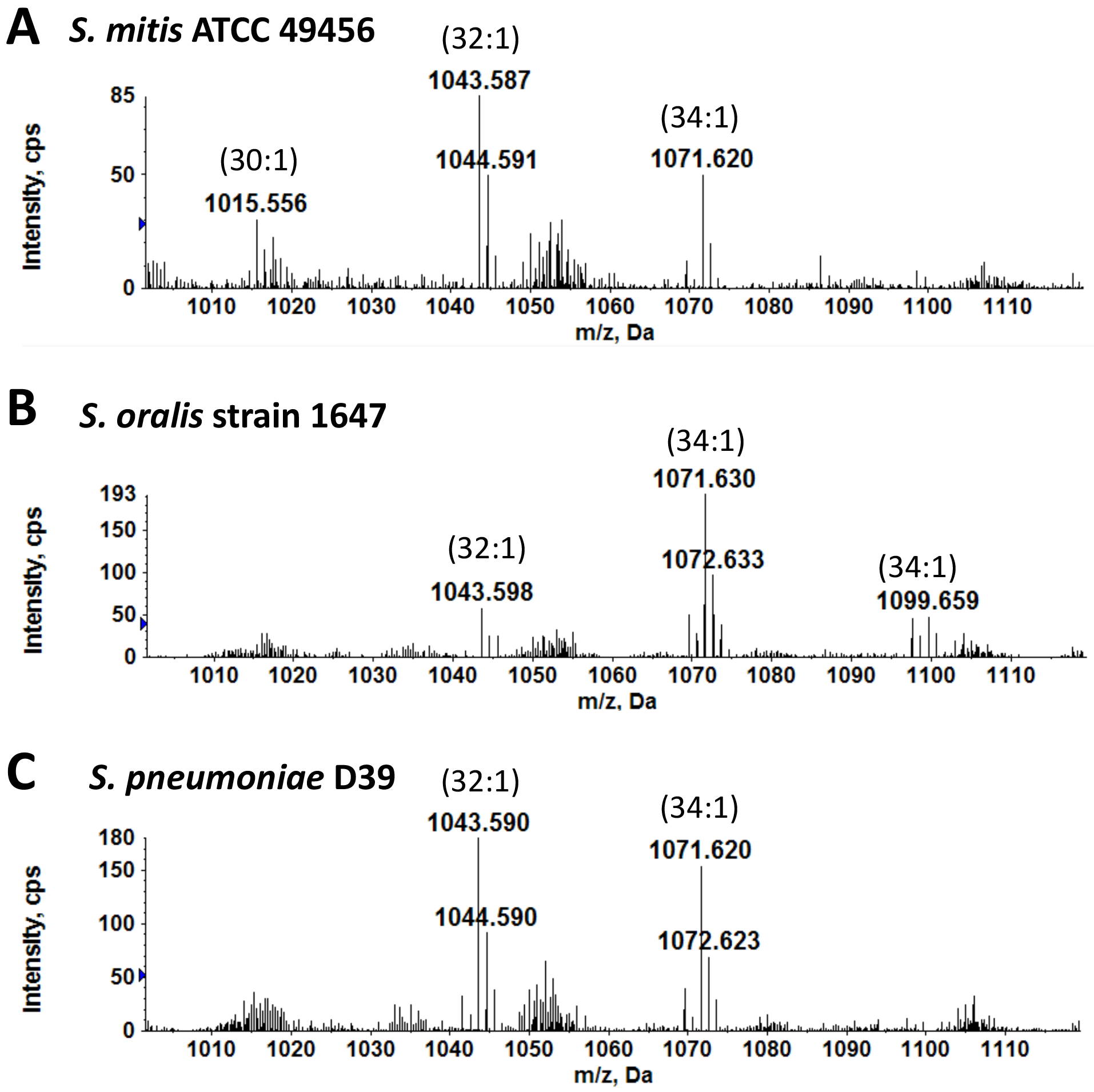

Supplement: FIG S2 [file msphere.01099-20-sf002.tif]

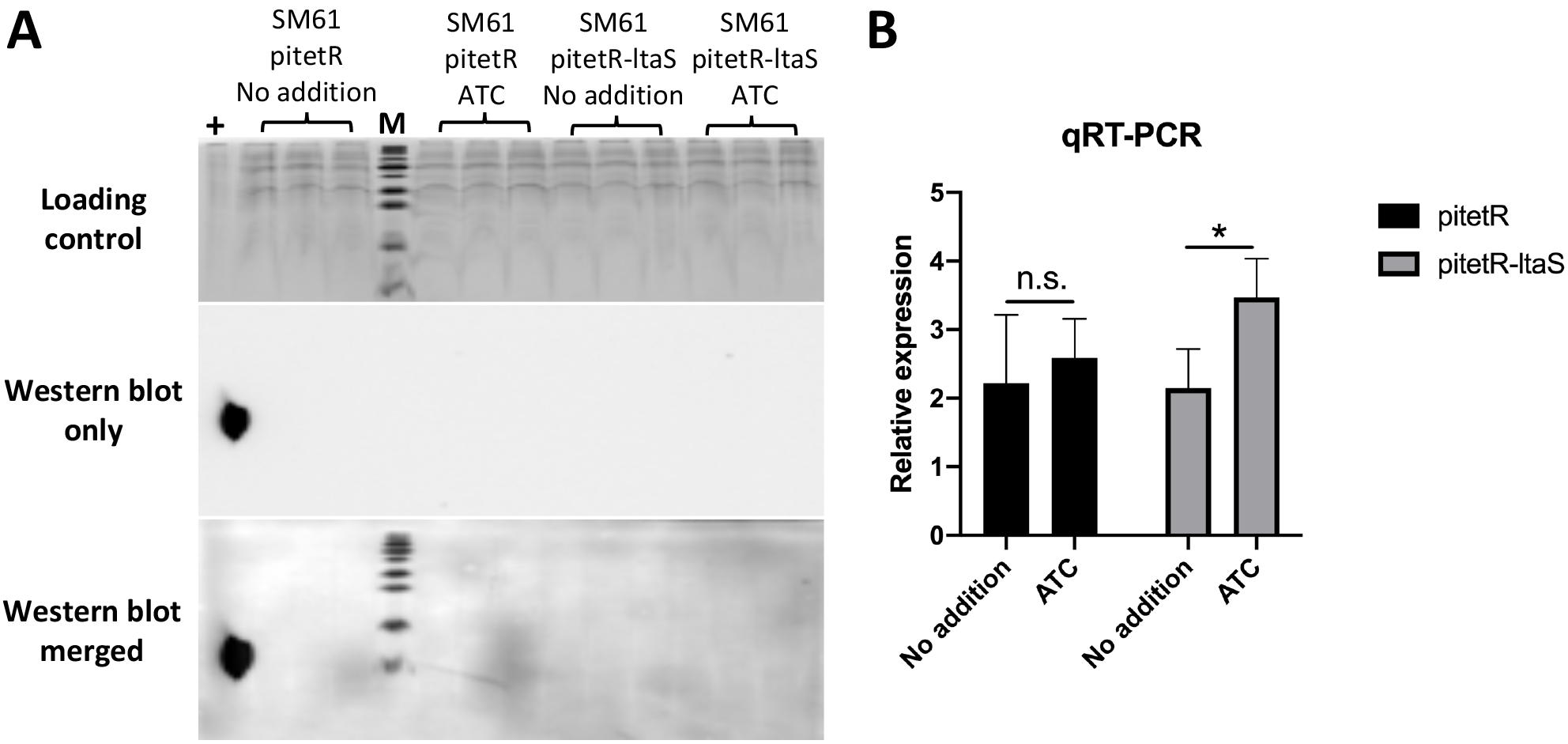

Supplement: FIG S3 [file msphere.01099-20-sf003.tif]

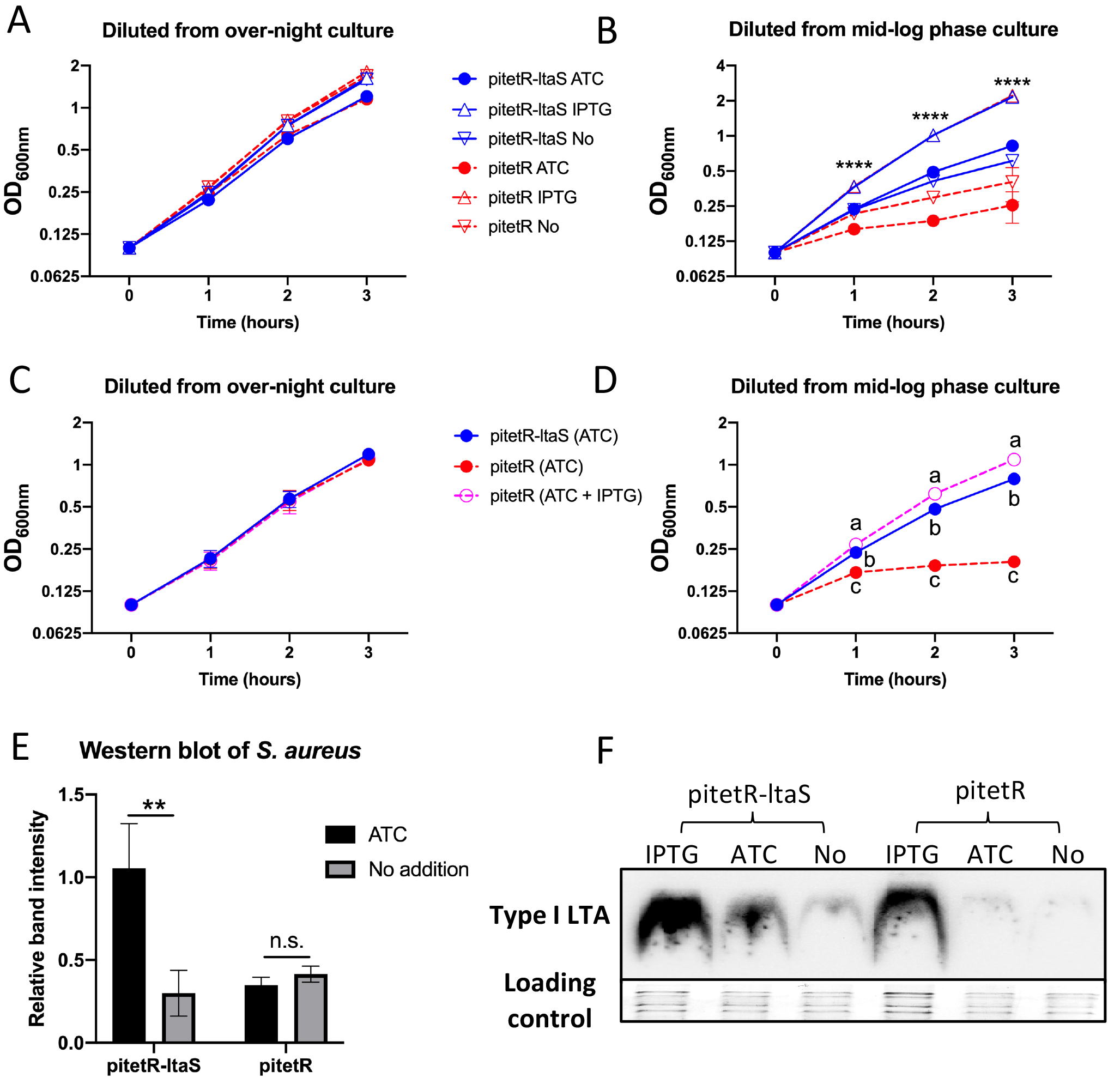

Supplement: FIG S4 [file msphere.01099-20-sf004.tif]

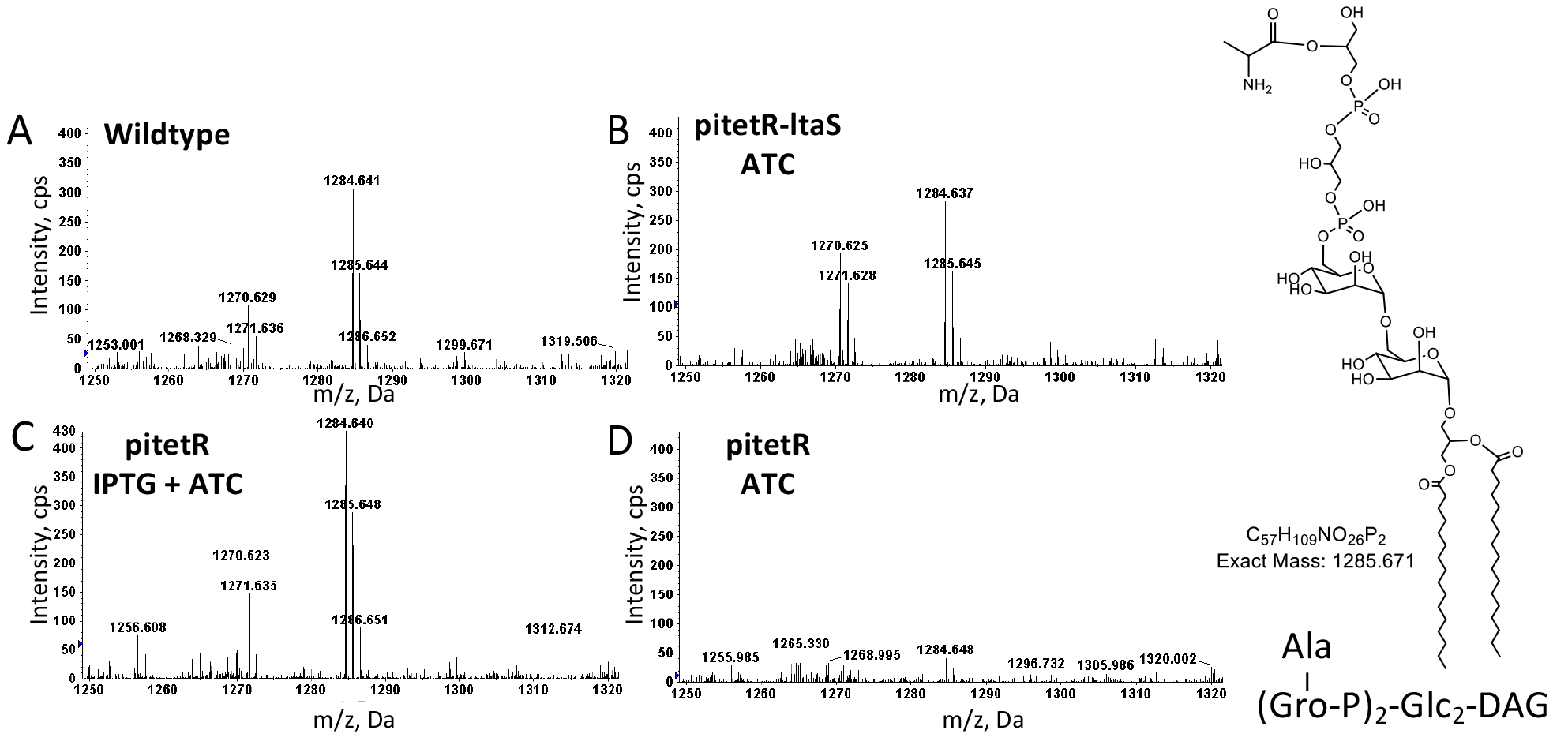

Supplement: FIG S5 [file msphere.01099-20-sf005.tif]

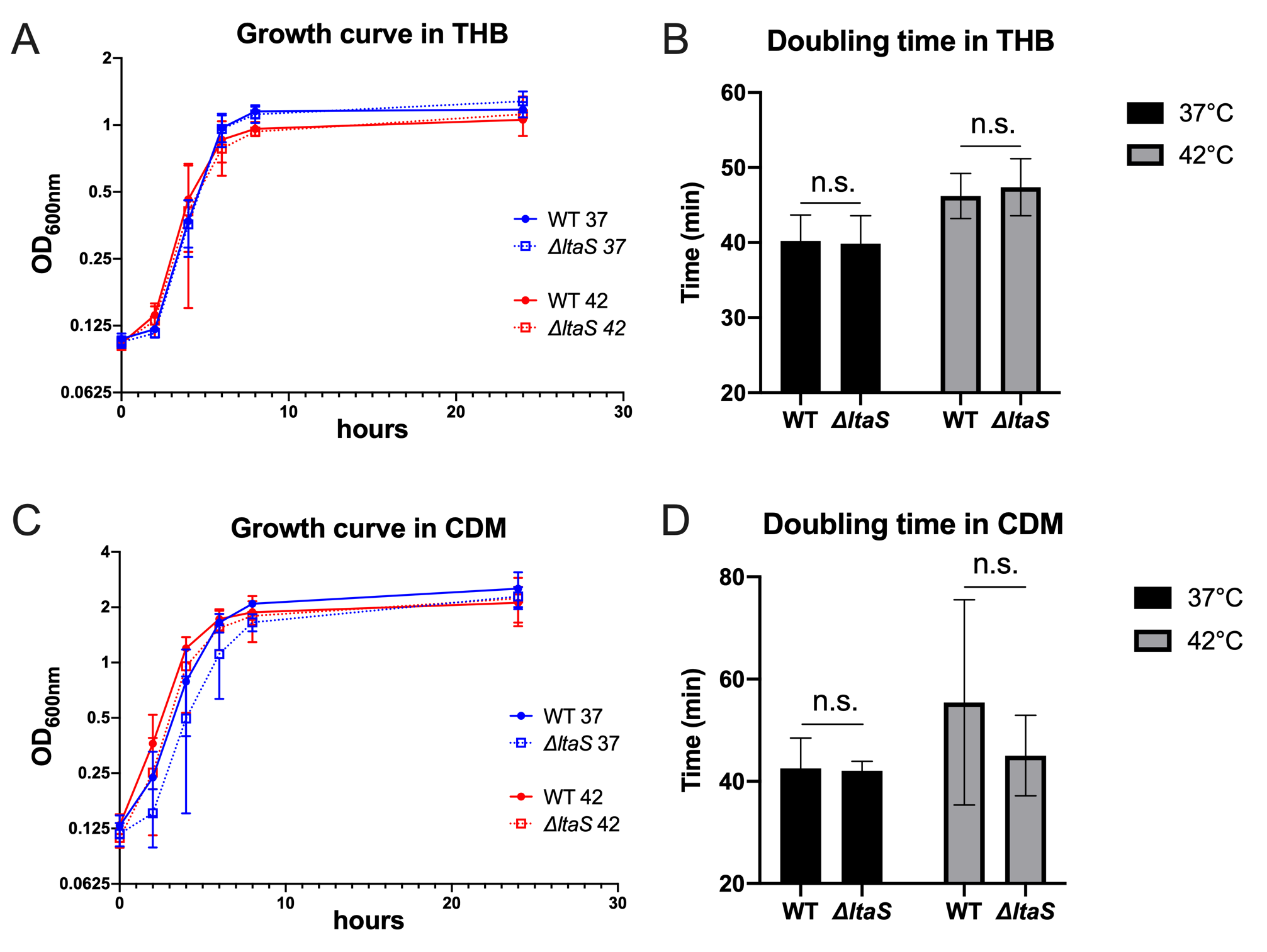

Supplement: FIG S6 [file msphere.01099-20-sf006.tif]
