# Supplementary material for: Streptococcus pneumoniae, S. mitis, and S. oralis Produce a Phosphatidylglycerol-Dependent, ltaS-Independent Glycerophosphate-Linked Glycolipid
Source: mSphere. 2021 Feb 24;6(1):e01099-20. doi: 10.1128/mSphere.01099-20 (PMC8544892; doi:10.1128/mSphere.01099-20)
Supplement: TABLE S1 [file msphere.01099-20-st001.pdf]

Table S1: E-test results of the wildtype and *ΔltaS* strain of *S. mitis* ATCC 49456 (SM61)

Strain	Antimicrobial Agent	MIC Median and Range (µg/mL)	S/R[a]
Wildtype	Ampicillin	0.023 (≤0.016−≤0.023)	S[b]
	Daptomycin	0.1575 (0.094−≤0.25)	NA[c]
	Vancomycin	0.5 (0.38−≤0.75)	S[b]
	Gentamycin	1.0 (0.75−≤1.0)	S[d]
	Linezolid	0.38 (0.38−≤1.0)	S[d]
	Cefazolin	0.19 (0.125−≤0.19)	S[b]
ΔltaS	Ampicillin	0.032 (0.023−≤0.032)	
	Daptomycin	0.142 (0.094−≤0.19)	
	Vancomycin	0.5 (0.5−0.5)	
	Gentamycin	1.0 (1.0−1.0)	
	Linezolid	0.5 (0.25−≤0.5)	
	Cefazolin	0.25 (0.19−≤0.25)	

[a] Abbreviations: S, susceptible; R, resistant; NA, not applicable.

[b] The European Committee on Antimicrobial Susceptibility Testing. Breakpoint tables for interpretation of MICs and zone diameters. Version 10.0, 2020. http://www.eucast.org

[c] No breakpoint has been established for daptomycin.

[d] CLSI. Performance Standards for Antimicrobial Susceptibility Testing. 30th ed. CLSI supplement M100. Wayne, PA: Clinical and Laboratory Standards Institute; 2020.
