# Supplementary material for: Streptococcus pneumoniae, S. mitis, and S. oralis Produce a Phosphatidylglycerol-Dependent, ltaS-Independent Glycerophosphate-Linked Glycolipid
Source: mSphere. 2021 Feb 24;6(1):e01099-20. doi: 10.1128/mSphere.01099-20 (PMC8544892; doi:10.1128/mSphere.01099-20)
Supplement: TABLE S2 [file msphere.01099-20-st002.pdf]

Table S2: Primers used in this research

Names	Sequences	Functions
S. mitis ATCC 49456 *cdsA* knockout fragment generation		
61cdsA_Up_F	CCAATCGTCTCCTCAAG	Amplify *cdsA* upstream fragment
61cdsA_Up_R_Xmal	ACGTCACCCGGGAAACAAGGTTCTTTTCTG	
61cdsA_Dwn_F_Xmal	ACGTCACCCGGGTTTCCAATCATGCACTTG	Amplify *cdsA* downstream fragment
61cdsA_Dwn_R	CTCGTTTGTTGCCATTTCC	
S. mitis ATCC 49456 *ltaS* knockout fragment generation		
YW1	CCATTCTCATTGACAACCGTATTAACC	Amplify *ltaS* upstream fragment
YW15	CCCTAGCGCTCTCACACAATTACTTCCTAAG	
YW6	ATGATCTGACTAGCTCTGATTTGGAG	Amplify *ltaS* downstream fragment
YW16	GCTACGGATCCAGAGTCGGCAGAAACCG	
YW13	ATTGTGTGAGAGCGCTAGGGACCTCTTTAGC	Amplify fragment with *ermB*
YW14	GCCGACTCTGGATCCGTAGCGGTTTTCAAAATTTG	
Constructing plasmid pitetR-ltaS		
pABG5-5	GGAAAGGGACCTCTCTTCCTAAAC	Linearize of pABG5
pABG5-3	GATAAAGGTATTGGTAAATAACAAA	
LtaS_F	GAGAGGTCCCTTTCCAGGAAGTAATTGTGTGAG	Amplify *S. mitis ltaS* coding region
LtaS_R	ACCAATACCTTTATCGAAGAGCATTTTTATTGTG	
YW55	GTGAGAATCAATTTTAACAAAATC	Linearize *ltaS* inserted pABG5
YW56	ACAATTACTTCCTGGAAAG	
Constructing plasmid pitetR empty		
YW58	GAGCATTTGAATTCACATGTTACCTCCTTTTGC	Linearize pitetR-ltaS. EcoRI cutting site is underlined.
YW59	GGTAACATGTGAATTCAAATGCTCTTCGATAAAGG	
Constructing plasmid pitetR-SAltaS		
YW72	AAAATAGCAAAAGGAGGTAAAATCATGAGTTCACAAAAAAAGAAAATTAG	Amplify *S. aureus ltaS* coding region
YW73	CCTTTATCGAAGAGCATTTGCCGAGTTCGTGTTTAAATATTATTTTTTAG	
Constructing plasmid pET-ltaS		

YW49	AACATGTATGGTGAGAATCAATTTTAACAAAATC	Amplify *S. mitis*
YW45	TCTCGAGTTATTGTGATTTTGATTCGG	*ltaS* coding region
